# Bridging immunogenetics and immunoproteomics: Model positional scanning library analysis for Major Histocompatibility Complex class II DQ in *Tursiops truncatus*

**DOI:** 10.1371/journal.pone.0201299

**Published:** 2018-08-02

**Authors:** Colette T. Dooley, Tatiana Ferrer, Heidi Pagán, Gregory M. O’Corry-Crowe

**Affiliations:** 1 Torrey Pines Institute for Molecular Studies, Port St. Lucie, Florida, United States of America; 2 Harbor Branch Oceanographic Institute, Florida Atlantic University, Fort Pierce, Florida, United States of America; Centro Cardiologico Monzino, ITALY

## Abstract

The Major Histocompatibility Complex (MHC) is a critical element in mounting an effective immune response in vertebrates against invading pathogens. Studies of MHC in wildlife populations have typically focused on assessing diversity within the peptide binding regions (PBR) of the MHC class II (MHC II) family, especially the DQ receptor genes. Such metrics of diversity, however, are of limited use to health risk assessment since functional analyses (where changes in the PBR are correlated to recognition/pathologies of known pathogen proteins), are difficult to conduct in wildlife species. Here we describe a means to predict the binding preferences of MHC proteins: We have developed a model positional scanning library analysis (MPSLA) by harnessing the power of mixture based combinatorial libraries to probe the peptide landscapes of distinct MHC II DQ proteins. The algorithm provided by *NNAlign* was employed to predict the binding affinities of sets of peptides generated for DQ proteins. These binding affinities were then used to retroactively construct a model Positional Scanning Library screen. To test the utility of the approach, a model screen was compared to physical combinatorial screens for human MHC II DP. Model library screens were generated for DQ proteins derived from sequence data from bottlenose dolphins from the Indian River Lagoon (IRL) and the Atlantic coast of Florida, and compared to screens of DQ proteins from Genbank for dolphin and three other cetaceans. To explore the peptide binding landscape for DQ proteins from the IRL, combinations of the amino acids identified as active were compiled into peptide sequence lists that were used to mine databases for representation in known proteins. The frequency of which peptide sequences predicted to bind the MHC protein are found in proteins from pathogens associated with marine mammals was found to be significant (p values <0.0001). Through this analysis, genetic variation in MHC (classes I and II) can now be associated with the binding repertoires of the expressed MHC proteins and subsequently used to identify target pathogens. This approach may be eventually applied to evaluate individual population and species risk for outbreaks of emerging diseases.

## Introduction

The Major Histocompatibility Complex (MHC) is responsible for the initial trigger in mounting an effective acquired immune response to pathogens in vertebrates. Genes of the MHC are understood to be under a selective pressure that is driven by the pathogens recognized by the encoded MHC proteins [1]. MHC genes are highly diverse and this polymorphism has been attributed to high rates of nucleotide substitutions in the peptide binding region (PBR) [2]. This phenomenon has found broad use in wildlife studies. This phenomenon has found utility in wildlife studies where population separation and environmental differences have been correlated with significant differences in MHC allelic frequencies [3]. Furthermore, positive selection has been found to shape diversity in MHC genes and MHC differentiation among populations, while a number of studies indicate that MHC may significantly influence fitness, either by affecting reproductive success or the survival of progeny to pathogen infections [4]. How MHC allelic diversity is able to translate to antigen recognition, and further to an effective response to specific pathogen communities, needs further study.

In wildlife populations, MHC diversity studies have mostly concentrated on variation in the MHC class II DQ and DR proteins [5]; the PBR of this dimer protein corresponds to exon 2 in the two genes, *DQA* and *DQB*. In cetaceans, studies have frequently compared variation in small regions of the *DQB* gene to that at neutral markers or analyzed the variation in amino acid (aa) residues expressed in the PBR [6–12]. For example, Vassilakos et al. [13] found patterns consistent with differential selection in regional populations of killer whale (*Orcinus orca*) and for 2 dolphin species (*Tursiops truncatus* and *Tursiops aduncus*). Furthermore, it was determined that both balancing and local positive selection pressures were important for defining the pattern of variation at the DQ locus. Cammen et al [14] characterized genetic variation at a short regions of *DQA* and *B* and *DRA* and *B* loci in *T*. *truncatus* in relation to exposure to harmful algal blooms in dolphins from central-west Florida and the Florida Panhandle. In the accompanying paper to the present study, Pagán *et al*. determined sequence variation for the entire exon-2 of both the *DQA* and *DQB* genes for bottlenose dolphins in the Indian River Lagoon (IRL), and adjacent Atlantic coastal populations of Florida. The study found that positive selection is influencing the genotypic variation within the PBR of both subunits of the dimer. Additionally these authors observed lower allelic diversity within the estuarine IRL compared to the coast. These results beg the question whether the lower diversity parallels a reduction in immune fitness for the estuarine population or whether selective forces render the remaining alleles capable of mounting immune responses with the same efficiency.

While such studies of MHC diversity are common [6–8,11,14–20], they have limited application to the assessment of immune fitness or the risks of infectious disease. Such investigations on MHC must ultimately be measured in terms of what they can tell us about the antigenic peptides, the different MHC variants that selectively bind, and which pathogens such peptides may be derived from. Only then can we interpret MHC diversity in terms of immune responses to specific pathogenic threats. If we can expand studies of MHC diversity to investigations of the entire MHC-peptide-pathogen axis, then we can develop a more holistic model of the factors influencing MHC diversity and finally begin to quantify risk that will assist in the management and recovery of wildlife populations.

Functional analysis, where changes in specific amino acids or motif changes within the PBR among different populations have been correlated to pathologies, has been used for some time in human studies to address these questions [21–25], and has recently been extended to wildlife populations [25,26]. Such studies, however, remain limited in wildlife species due to a lack of resources and the challenges of working on such species. Multiple wildlife populations and species are often involved requiring the development of multiple assays. Furthermore, binding peptides are seldom available for wildlife genotypes further hampering assay development. Finally, functional analysis typically does not explore the entire peptide-pathogen landscape.

Fortunately, studies on human MHC II (i.e., HLA II Human Leukocyte Antigen) have made great strides in this area and there are several algorithms for predicting peptide binding affinities for different MHC genotypes and generating databases of individual peptides and their binding affinities. Combinatorial libraries have also been used to directly assay the binding affinities of millions of peptides for both MHC class I and II in humans [27–29]. However, the latter studies generally require the use of a radiolabeled binding peptide to generate a binding assay. For those working on wildlife populations, sufficient resources are not generally available to develop specific binding assays and often impractical since multiple assays would be need to be developed in order to cover the number of different species and/or populations under study.

In this study, we develop a method to efficiently characterize the MHC-peptide-pathogen axis and test it against empirical data on MHC II diversity in a number of cetacean species. Using a combined immunogenetic and proteomic approach, we present a novel method that combines (a) predicting the binding affinities of large numbers of peptides to different MHC II protein variants with (b) reverse modeling of positional scanning library analysis to determine the most active peptides for the different MHC proteins. Bioinformatic tools (c) were then used to search protein databases for microorganisms that host such peptides. Finally, these microorganism databases were (d) explored for recognized and potential cetacean pathogens. We have successfully achieved our goal to develop a method that enables researchers to assess the consequences of mutations at the genetic level on the MHC binding landscape and thus predict the role of MHC genetic diversity on pathogen recognition.

## Results

### Peptide binding affinities for DQ alleles from *Tursiops truncatus*

**Fig 1:**
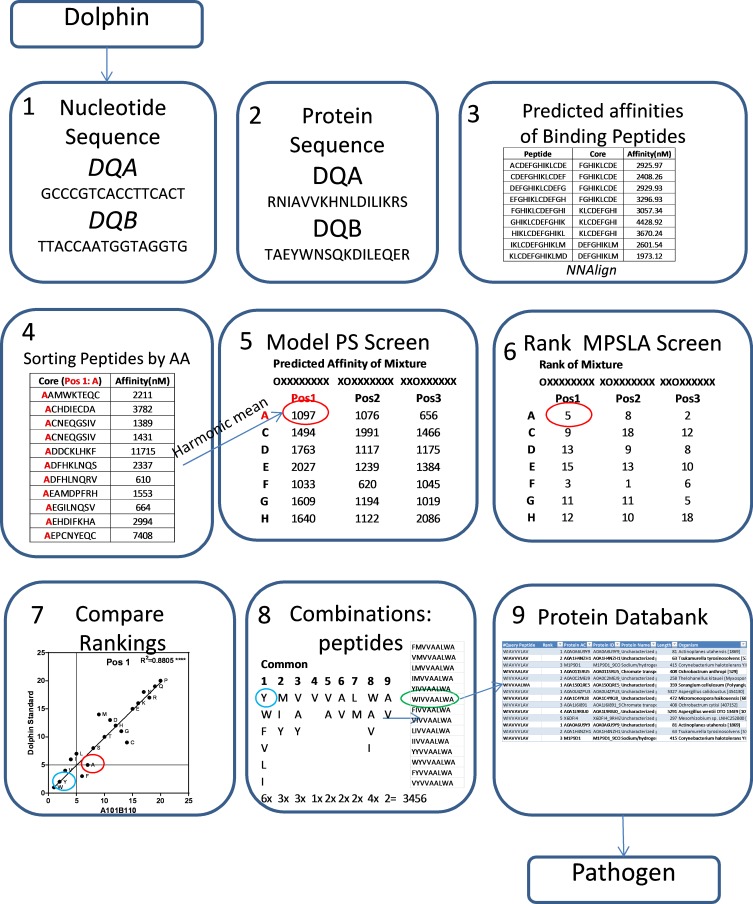
Method for model positional scanning library analysis (MPSLA). Nine steps that take the researcher from genetic sequence data, through MHC binding analysis, to protein and pathogen prediction.

**Fig 2:**
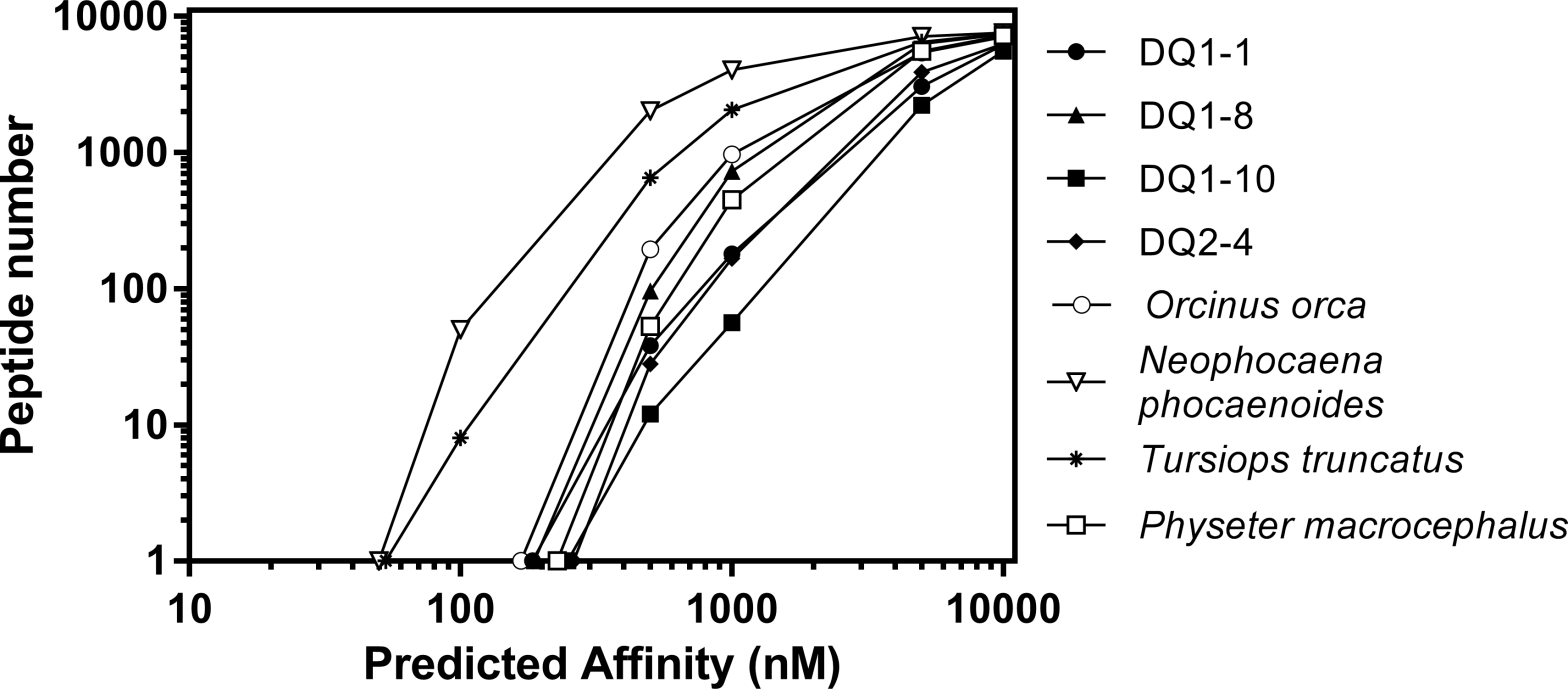
Predicted binding affinities of peptide sequences derived from proteins encoded by cetacean MHC II DQ alleles. Binding affinities of 7,634 peptide sequences predicted by *NNAlign* were compared by counting the number of peptides below 100, 500, 1000, 5000 and 10,000nM thresholds. The algorithm was supplied with a 7,647 amino acid sequence and the DQA and B protein sequences from cetaceans (killer whale, sperm whale, finless dolphin) obtained from Genbank and from bottlenose dolphins in the Indian River Lagoon (IRL) and adjacent Atlantic coast (ATL) (DQ1-1; Protein derived from DQA 1*01 DQB 1*01 DQ1-8; Protein derived from DQA 1*01 DQB 1*08 DQ1-10; Protein derived from DQA 1*01 DQB 1*10 and DQ2-4; Protein derived from DQA 1*02 DQB 1*04).

To assess the effect of variations in DQ genes of dolphins on the binding capacities of the expressed proteins, nucleotide sequences of exon 2 were translated and inserted into their representative DQA and DQB protein sequences and subsequently used to predict binding affinities of a series of independently generated peptides using the neural network-based method *NNAlign* [30]. The process was initiated by generation of a sequence of 7,647 amino acids of near equivalent representation of the 20 amino acids. The aa (amino acid) list (S1 Fig) was supplied to the server (NetMHCIIpan 3.1), along with the sequences for the DQ dimer proteins. For each DQ protein, *NNAlign* generated a series of 7,634 13mer peptide sequences, a length typically bound by MHC receptor proteins and predicted both the binding affinity of the 13mer, and identified a 9mer core, (steps 1–3 in Fig 1). The peptide series and their predicted binding affinities, were generated for each of four DQ proteins (derived from DQA and DQB alleles and genotypes found within the Florida Atlantic coastal and estuarine populations by Pagán *et al*., see Table 1 and Materials and Methods). Each of the dolphin DQ proteins were compared for their capacity to bind peptides, and plotted in terms of number of peptide sequences with affinities below 100, 500, 1,000, 5,000 and 10,000 nM (Fig 2). Similar plots were also generated for peptide affinities generated from a representative DQ protein bottlenose dolphin (used as standard) and DQ proteins from three other cetacean species: killer whale (*Orcinus orca*), finless porpoise (*Neophocaena phocaenoides*), and sperm whale (*Physeter macrocephalus*) (sequences were obtained from GenBank and accession numbers are given in the Methods section). Comparison of the affinity plots predict weaker binding affinities overall for DQ protein variants found in dolphins from the IRL as compared to our ‘standard’ (i.e., GenBank) bottlenose dolphin DQ protein, nevertheless DQ proteins from Floridian dolphins would exhibit strong affinities (<300nM) for a small number of peptides (Fig 2). The binding affinity profile for DQ1-8 was predicted to exhibit the strongest binding affinities of the four DQ proteins from the IRL, and was similar in profile to the affinities observed for DQ proteins derived from killer and sperm whales.

**Table 1:** Frequency of selected alleles for bottlenose dolphin from Pagán et al.

A.		**ATL**	**ML**	**NIRL**	**SIRL**
		**%**	**%**	**%**	**%**
	***DQA STD***	18	5	0	7
	DQA 1*01	16	36	42	45
	DQA 1*02	23	34	50	45
**Alleles**		n = 44	n = 44	n = 36	n = 60
	DQB 1*01	20	6	0	7
	DQB 1*04	10	31	47	45
	DQB 1*08	3	6	6	17
	DQB 1*10	13	31	38	28
		n = 30	n = 36	n = 34	n = 58
B.		**ATL**	**ML**	**NIRL**	**SIRL**	**Protein**
	DQA 1*01 DQB 1*01	1	—	—	2	DQ1-1
**Haplotypes**	DQA 1*01 DQB 1*08	—	1	—	—	DQ1-8
	DQA 1*01 DQB 1*10	—	3	3	1	DQ1-10
	DQA 1*02 DQB 1*04	—	2	5	5	DQ2-4

DQA and DQB peptide binding regions (exon 2) were genotyped in bottlenose dolphins from Florida Mosquito Lagoon (ML), North Indian River Lagoon (NIRL), and South IRL (SIRL) as well as the adjacent Atlantic coast (ATL). (A) Frequency of allele in sample population (n) expressed as a percentage. (B) The DQA/DQB haplotypes were determined from homozygous individuals or inferred from heterozygotes. STD; standard.

**Fig 3:**
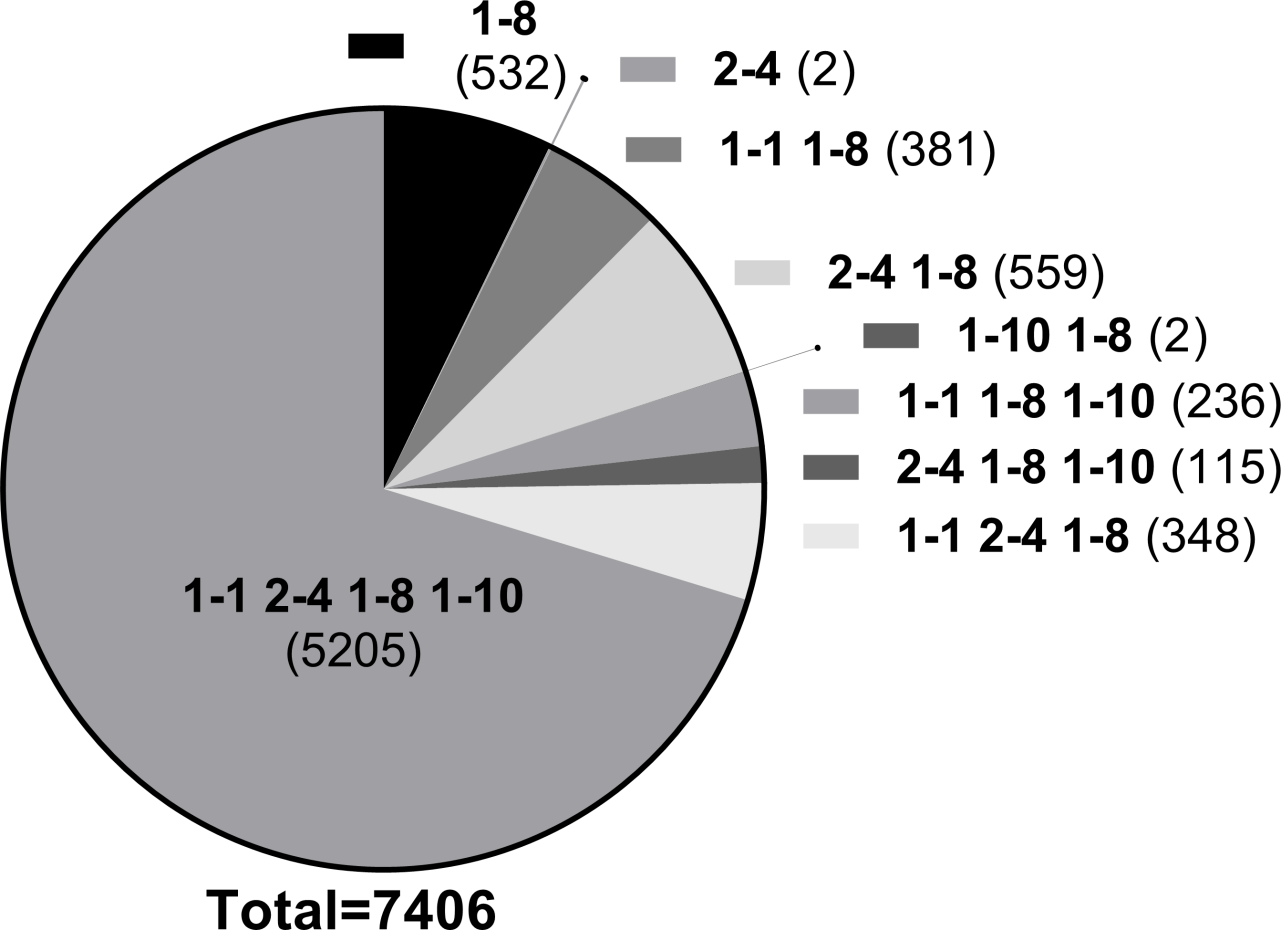
Distribution of active sequences derived from dolphin DQ proteins from the IRL. Sequences with a binding affinity below 10,000nM for each of the datasets for the four proteins (DQ1-1, DQ1-8, DQ1-10 and DQ2-4) from dolphins in the Indian River Lagoon and adjacent Atlantic coast were compiled and the frequency of sequences identified uniquely binding to one protein or shared by 2, 3 or all 4 proteins is shown.

The frequency of individual 13-mer peptide sequences in binding affinity lists generated for four IRL-ATL DQ proteins was also examined. Peptide sequences (limited to those with binding affinities likely to have meaningful activity i.e. <10μM) that had high binding affinities for just one or for 2, 3 or all 4 DQ proteins were totaled (Fig 3.). A high level of sequence overlap was observed (i.e. peptide sequences identified as active in all four proteins). This was not altogether surprising as the DQ proteins derived from these haplotypes differ by only a few amino acids, and not all of these differences were found in the binding pockets, the site most likely to affect binding affinity. However, we did identify peptide sequences that were predicted to bind to some DQ haplotypes and not others suggesting that the DQ proteins are likely to have distinct binding landscapes. For example, 532 peptides had high binding affinities for DQ1-8 and not for any of the other proteins. These analyses offered new insights into peptide binding in dolphin DQ proteins. They also highlight, however, the necessity to address how mutations in the binding pockets of these DQ genotypes influence the total binding landscape and moreover, whether such changes have a positive or negative influence on the MHC protein’s capacity to recognize pathogens.

### Model positional scanning library analysis of predicted binding affinities for DQ alleles

MHC II proteins recognize a large population of binding peptides and moreover, unlike other binding proteins, the binding sequences capable of triggering immune responses are not as tightly correlated with affinity [31], making it challenging to identify likely natural sources. There has been no method to rapidly determine whether MHC proteins encoded by closely related alleles differ significantly in their binding landscape and certainly none that would indicate whether such differences have a positive or negative affect on the organism’s ability to thrive in its environment.

Combinatorial libraries are composed of a series of mixtures that contain all possible peptide sequences for a particular peptide length and have been in use for over 25 years [32–34]. A Positional Scanning (PS) library is composed of several arrangements of the same peptide sequences, such that each arrangement addresses a single position in a peptide of a defined length. For example, for a PS library of a peptide with six amino acids there are six arrangements OXXXXX, XOXXXX, XXOXXX, XXXOXX, XXXXOX, XXXXXO, (where O represents a defined amino acid and X represents a mixture of amino acids) of 20 mixtures; one for each L amino acid. Each mixture contains (1X20X20X20X20X 20 = 64,200,000 peptides). Screening of a PS library identifies the most active amino acid(s) at each position and the combination of active amino acids generates the peptide sequences active in the assay (for a complete review of mixture based libraries generation and their use see [35]). Thus, combinatorial libraries are an ideal means to investigate the peptide binding landscape for MHC proteins and this method has been used for human MHC proteins [27,28]. However, screening of combinatorial libraries requires setting up of a binding assay and prior identification of an active sequence. Such resources are not often available for those studying other vertebrates and are not practical for the study of multiple genotypes of MHC proteins. In order to analyze the peptide binding profiles for the four cetacean DQ genotypes, we retroactively constructed a model PS Library (steps 4 and 5 in Fig 1). We used the affinities of the 7,634 sequences generated by *NNAlign* to model a 9-mer core combinatorial library. Since the activity of a mixture is driven by the affinities of its most active components and it is not diluted by its weak or non-active components, a mixture’s activity is calculated by using the harmonic mean of the combined affinities of components of the mixture [36]. The nonamer core of the 13-mer sequences generated in *NNAlign* and their corresponding affinities were used to generate “calculated mixture affinities”. For example, all sequences with Alanine (A) at position 1 were extracted from the *NNAlign* list and the corresponding affinities were used to calculate the harmonic mean and therefore the activity of the hypothetical mixture (AXXXXXXXX). A physical positional scanning 9-mer library would actually contain trillions of peptides in such a mixture; however the vast majority of the peptides are not likely to have any activity. Since the activity is governed by the harmonic mean and we are using many peptides with predicted activities we can reasonably assume “calculated mixture affinity” will reflect the activity of a physical mixture.

**Fig 4:**
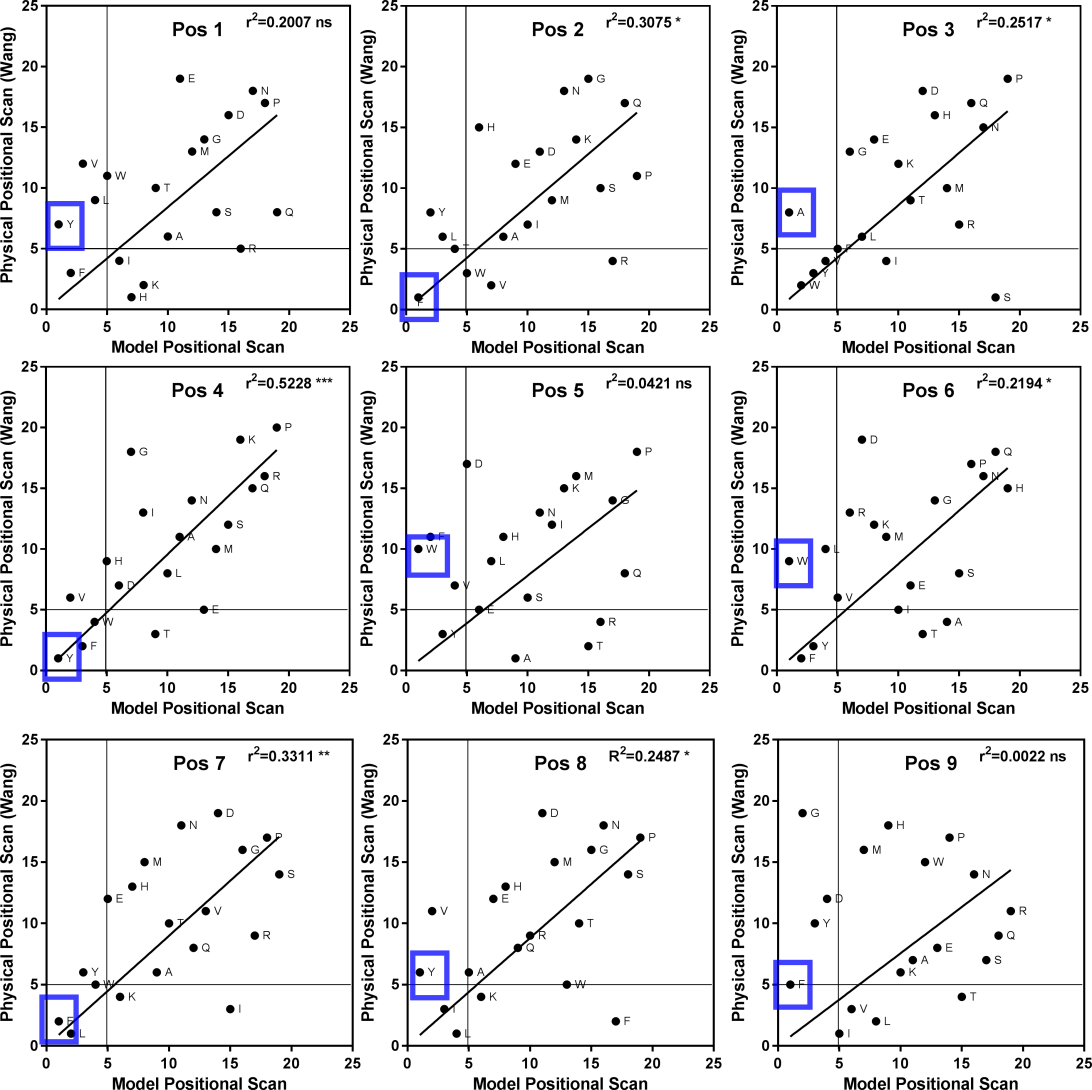
Comparison of amino acid ranking from physical and model positional scanning libraries. Binding affinities for the (19 x 9 = 171) mixtures obtained from the screening of the physical protein or modeled protein encoded by HLA-DP2 (*Hs*DPA1*0103, *Hs*DPB1*0201) were ranked from 1–19, where 1 represents the lowest value and therefore highest binding affinity. Correlations were performed on scatterplots, Coefficients of determination (r^2^) derived from Pearson coefficients (r) are recorded in upper right corner.

To test our hypothesis that we could accurately model a library screen, we compared our model PS to a physical combinatorial screen for the human HLA DP2 receptor protein (26). The harmonic mean was calculated for each of the 20 x 9 = 180 “calculated mixtures” to generate a Model Positional Scan 9-mer library (S1 Table). To compare the different positional scanning libraries, the mixtures at each of the 9 positions were ranked and scatterplots were generated from the two ranking sets (Steps 6 and 7 in Fig 1). The calculated affinities for the 171 mixtures of the model PS (19 x 9 = 171, no cysteine, see Methods) with their rankings from 1–19 where 1 corresponds to the lowest value (i.e. most active mixture) are presented in Table 2. Scatter plots of the rankings of the physical versus the model libraries revealed low but significant correlations in 6 of the 9 positions (Fig 4). There was some difficulty in replicating data from the physical library [28] as it was based on a 13-mer peptide with di-alanine at the proximal and terminal ends (AAXXXXXXXXXAA), this could not be exactly replicated using *NN-Align*. However this method is an improvement on a molecular docking approach by Patronov *et al*. [37] that used 247 modelled peptide-DP2 complexes (DS-QMnap) when we ranked data from this study and plotted them against data for physical library only 3 of the 9 positions yielded significant correlations (Table 3), suggesting that the method we describe is more likely to resemble a physical library screen.

**Table 2:** Model positional scanning library and ranking for MCH II DP.

A	Position (calculated affinities nM)									B	Position (aa)								
AA	1	2	3	4	5	6	7	8	9	Rank	1	2	3	4	5	6	7	8	9
**A**	835	534	**230**	725	430	878	592	385	551	**1**	**Y**	**F**	**A**	**Y**	**W**	**F**	**F**	**Y**	**F**
**D**	1818	620	745	410	328	601	885	670	414	**2**	F	Y	W	V	F	W	L	V	G
**E**	1018	615	540	898	388	795	421	424	744	**3**	V	L	Y	F	Y	Y	Y	L	Y
**F**	358	**254**	308	338	217	**264**	**233**	853	**310**	**4**	L	V	V	W	V	L	E	A	D
**G**	1277	802	483	449	929	862	1042	803	340	**5**	W	H	F	D	D	D	W	I	I
**H**	655	422	781	414	408	1118	543	449	450	**6**	I	T	L	H	E	V	K	E	V
**I**	629	635	556	475	576	731	920	400	435	**7**	H	W	G	G	L	R	H	K	H
**K**	866	732	645	1336	683	716	479	428	528	**8**	A	A	E	I	H	K	M	H	L
**L**	492	342	462	751	395	399	357	368	466	**9**	K	E	I	T	A	I	A	R	M
**M**	1058	677	875	792	707	803	560	679	498	**10**	T	D	K	A	S	E	T	Q	K
**N**	2110	659	1187	850	591	889	752	831	1561	**11**	E	I	T	L	I	M	N	D	A
**P**	2042	2809	1931	2024	1377	847	1702	1634	990	**12**	M	N	D	M	N	T	Q	M	W
**Q**	2250	1997	1066	1247	1138	944	762	623	1648	**13**	G	M	H	N	K	P	D	T	E
**R**	1775	1244	931	1678	869	712	1062	603	2127	**14**	R	K	M	E	M	G	V	W	T
**S**	1805	929	1322	1005	563	917	1893	1544	1625	**15**	S	G	R	S	T	A	I	G	P
**T**	926	430	650	713	801	816	705	712	959	**16**	D	S	Q	Q	R	N	G	N	N
**V**	476	403	278	281	317	660	906	330	443	**17**	P	R	N	K	G	S	R	F	S
**W**	552	445	262	377	**201**	267	436	734	659	**18**	N	Q	S	R	Q	Q	P	S	Q
**Y**	**220**	305	263	**242**	247	280	384	**256**	408	**19**	Q	P	P	P	P	H	S	P	R

(A) The calculated binding affinities for mixtures (nM) derived from the sequences affinities generated by NetMHCIIpan 3.1 for each of the 19 amino acids at nine positions of the core binding peptide for the human HLA DP2 protein. (B) The amino acids (single letter code A-Y) defined in the mixtures are sorted by mixture affinity. Mixtures with the lowest value and highest affinity (those ranked 1) are in blue on both sides, those ranked 1–5 are in gray on right.

**Table 3:** Coefficients of determination (r^2^) derived from pearson correlation coefficients (r) from scatterplots of amino acid rankings.

Scatterplots of amino acid rankings			Position								
**A**			**1**	**2**	**3**	**4**	**5**	**6**	**7**	**8**	**9**
*Hs*DP											
Physical Library	v	MPSLA	0.2255	0.2690	0.2639	0.5276	0.0428	0.1671	0.3017	0.2233	0.0040
			*	*	*	***	ns	ns	*	*	ns
Physical Library	v	DS-QMnap	0.3491	0.2532	0.1682	0.0052	0.0018	0.0808	0.2615	0.0257	0.0021
			**	*	ns	ns	ns	ns	*	ns	ns
DS-QMnap		MPSLA	0.5042	0.4159	0.1505	0.0025	0.0562	0.1266	0.0182	0.0439	0.0110
			***	***	ns	ns	ns	ns	ns	ns	ns
**B**											
DQ 1–10	v	DQ 1–8	0.6741	0.7193	0.7607	0.628	0.5470	0.6840	0.7792	0.8195	0.8442
DQ 1–10	v	DQ *Neo*. *ph*.^*a*^	0.8086	0.5813	0.7142	0.6643	0.2359	0.2187	0.3600	0.6068	0.6643
DQ 1–1	v	DQ *Neo*. *ph*.^*a*^	0.6256	0.5452	0.2330	0.3422	0.4201	0.6424	0.3404	0.5586	0.4640
DQ 1–1	v	DQ *Phy*. *ma*.^*b*^	0.6717	0.5541	0.5744	0.7193	0.7581	0.8359	0.7872	0.9004	0.8581

^a^Neo.ph; Neophocaena phocaenoides

^b^Phy.ma; Physeter macrocephalus

Statistical significance as defined by Graphpad;

P values 0.1234 (ns), 0.0332(*), 0.0021(**), 0.0002 (***) and <0.00001(****).

A. Coefficients of determination (r^2^) were derived from Pearson coefficient values for each of the 9 positions and statistical significance. Correlations were performed for amino acid ranking values obtained from human HLA-DP alleles using 3 methods; Physical Positional Scanning Library, Model Positional scanning library (MPSLA) and Model of amino acid preference (DS-QMnap). B. DQ proteins from the Indian River Lagoon that differed significantly from the standard DQ (correlations generated low Pearson coefficient values) were found to have a higher degree of relatedness (higher Pearson coefficient values) when compared to 2 other cetacean species (Yangtze finless porpoise and sperm whale).

**Fig 5:**
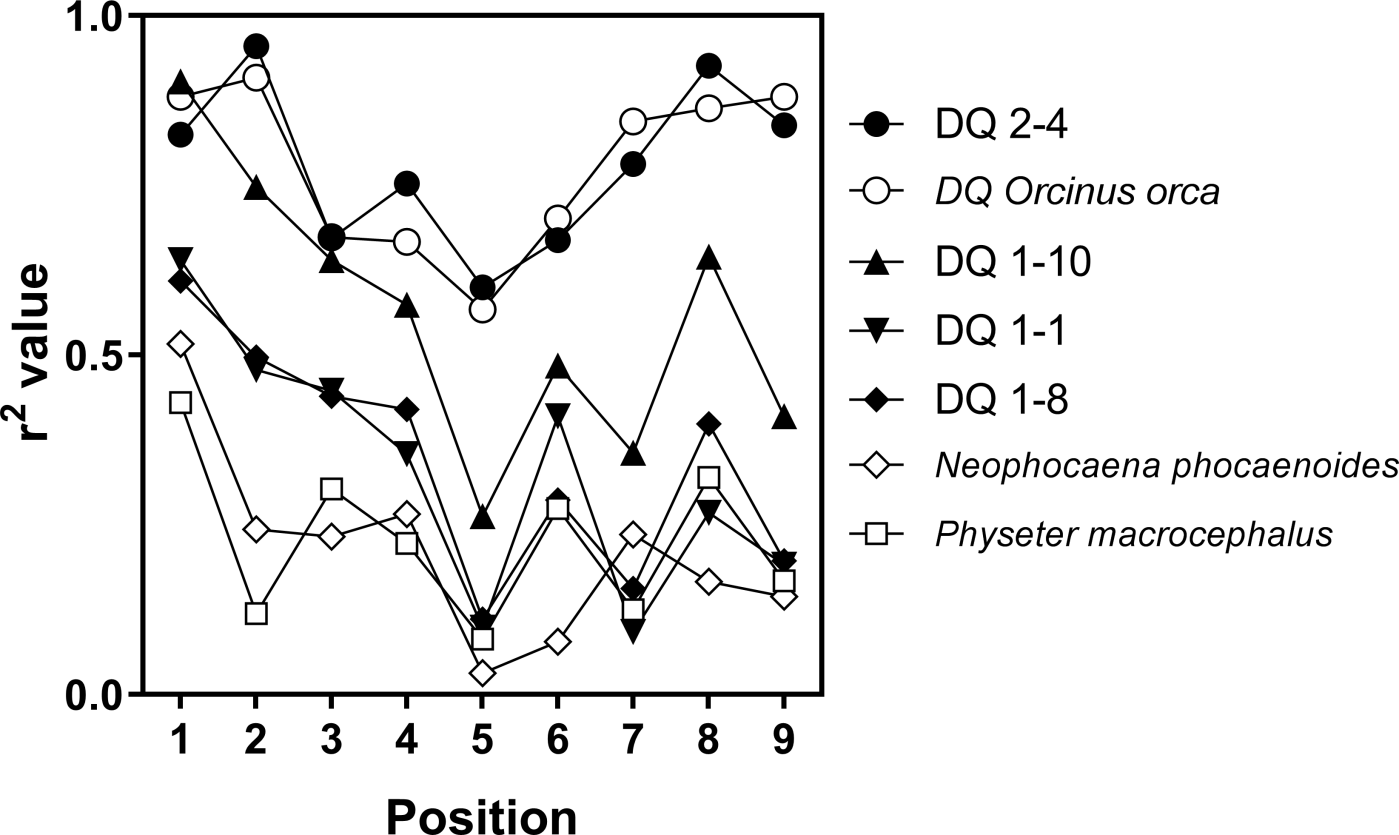
Comparison of rankings for dolphin (standard) to four IRL dolphins and 3 other cetaceans. Correlations were performed on scatterplots of amino acid ranking obtained from our standard DQ protein of Bottlenose dolphin (*Tursiops truncatus*), with each of the four proteins found in the IRL (DQ1-1, DQA 1–8, DQA1-10 and DQ2-4). Correlations were also performed comparing the amino acid ranking from DQ proteins for standard bottlenose dolphin to those of killer whale (*Orcinus orca*), Yangtze finless porpoise (*Neophocaena phocaenoides*) and sperm whale (*Physeter microcephalus*). Coefficients of determination (r^2^) derived from Pearson coefficients (r) are plotted for each of the 9 positions of the MPSLs.

Since comparisons within the same predictive system should cancel out system bias, we prepared model PS libraries from the predicted affinities of peptides that bind to 4 DQ proteins from the Florida dolphin populations, a DQ derived from sequencing data for dolphin (standard) and for DQ proteins for 3 other cetacean species (S1–S8 Tables). The ranking values for these matrices were used to generate scatterplots against rankings derived from the dolphin standard. Correlation coefficients were calculated for each of the nine positions (Fig 5). Data from the analysis indicated that rankings for the protein DQ2-4 from dolphin in the IRL has a very high level of correlation with the dolphin reference standard (all positions exhibited high values for the Coefficient of determination (r^2^) derived from Pearson coefficient values r, (r^2^ values above 0.7). Rankings for DQ2-4 were also very closely correlated with data for DQ from killer whale. IRL derived proteins DQ 1–1 and DQ 1–10 exhibited similar patterns when correlated with the standard dolphin DQ, and Pearson coefficients were greater than 0.7 for all 9 positions when the two were compared directly. This would suggest that, although there are 6 amino acid changes between the DQB in these two proteins, the peptides recognized by the proteins are very similar; however, the binding affinities may be different as suggested by binding affinity plots (Fig 4). Similarly, the standard DQ, DQ2-4 and DQ from killer whale should all recognize a comparable array of peptide sequences. DQ proteins from finless porpoise and sperm whale have less in common with the DQ standard (low Pearson coefficients), thus are more likely to recognize a very different set of peptides sequences. It should be noted that only the confirmed haplotypes (i.e., DQA and DQB alleles from homozygous individuals, DQ1-8, DQ1-10 and DQ2-4) are representative of genuine DQ peptide binding regions. Data from WGS projects produce a single consensus from diploid organisms and thus may not correctly present true heterozygous positions [38]. Likewise, unphased DQA and DQB alleles from heterozygous individuals represent only probable haplotypic combinations (e.g. standard DQ, DQA1-1, killer whale, sperm whale and finless porpoise). Nevertheless, these data provide the groundwork for establishing the utility of these methods, and they allow for initial cross-species comparisons and predictions that greatly exceed the scope of standard wildlife MHC genotyping projects.

### Predicted peptide sequences from MPSLA and their association with proteins and pathogen sources

While the model positional scanning method presented here sheds new light on the relationship between the peptide binding capabilities of closely related MHC II protein variants, the true value of such analyses comes from the ability to predict active binding sequences for MHC II and more importantly linking them to the protein sources and the pathogens that produce them. Since MHC II proteins can bind a vast number of peptide sequences, and binding affinity is not always a strong predictor for functional activity, it is nearly impossible to know which binding peptides are likely to be of biological significance. To overcome this impasse we have used the amino acid rankings from Model PS libraries for 3 IRL DQ proteins to select amino acids preferred by particular DQ proteins and used highest ranking amino acids held in common to both proteins to predict peptide sequences of biological significance. The amino acids determined to be of significance (i.e., ranked highly in one or both of the DQ proteins) were used in combinatorial fashion to generate a new list of 9-mer peptide sequences that in turn were used to mine the protein databases (UniProtKB through the Protein Information Resource (PIR) [39]). The searches generated tables listing matching sequences, the proteins where they occur, and the organism from where the protein is derived.

**Fig 6:**
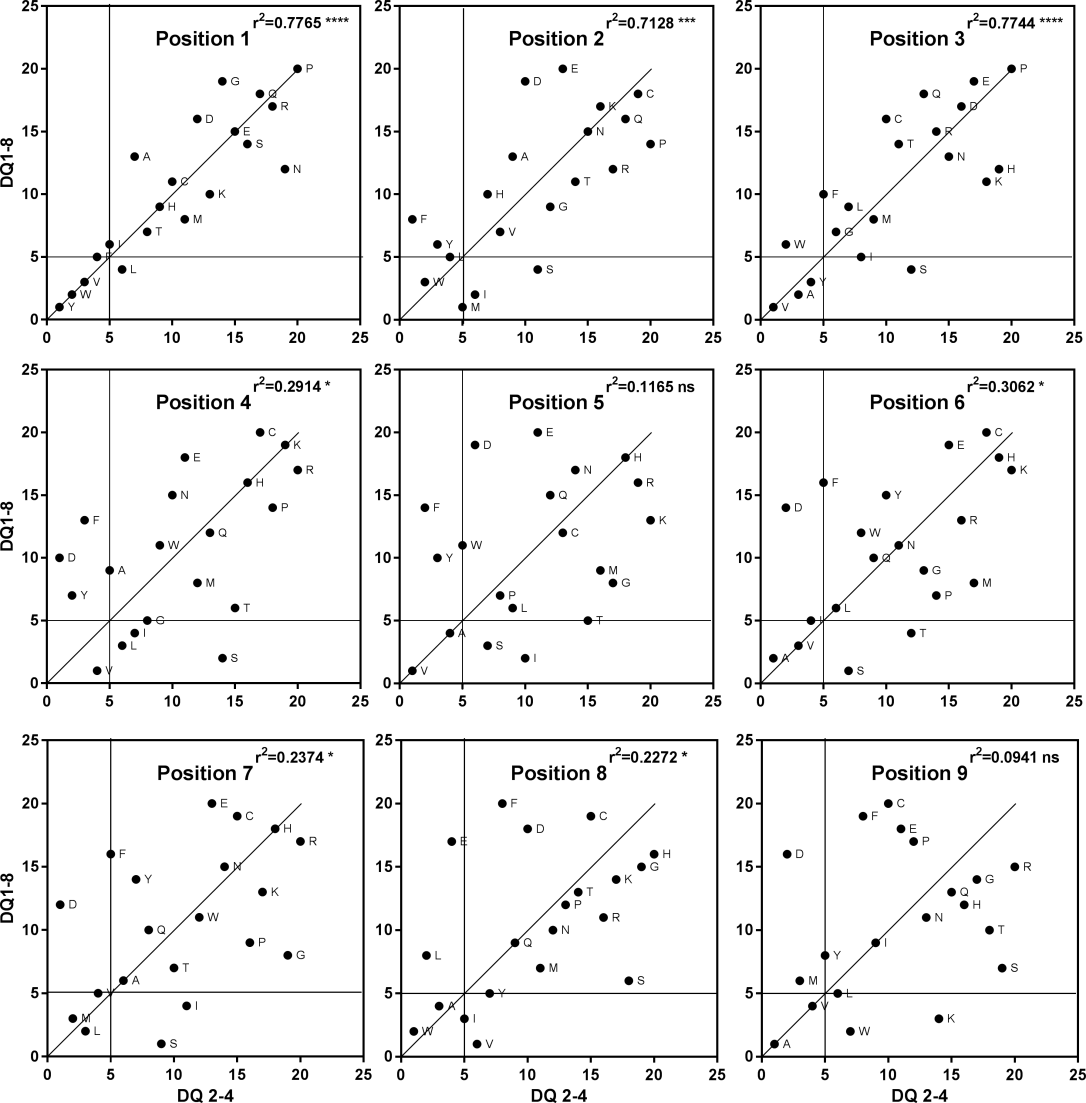
Comparison of amino acid ranking from MPSLs derived for DQ1-10 and DQ2-4 in dolphin. Predicted binding affinities for the (20 x 9 = 180) mixtures obtained from the model positional scanning libraries derived from proteins encoded by dolphin alleles DQA1*01DQB 1*10 (DQ1-10) and DQA1*02 DQB1*04 (DQ2-4) were ranked from 1–20, where 1 represents the lowest value and therefore highest binding affinity. Correlations were performed on scatterplots, coefficients of determination (r^2^) derived from Pearson coefficients (r) are recorded in upper right corner. Vertical and horizontal lines demark amino acids ranked below 5.

**Fig 7:**
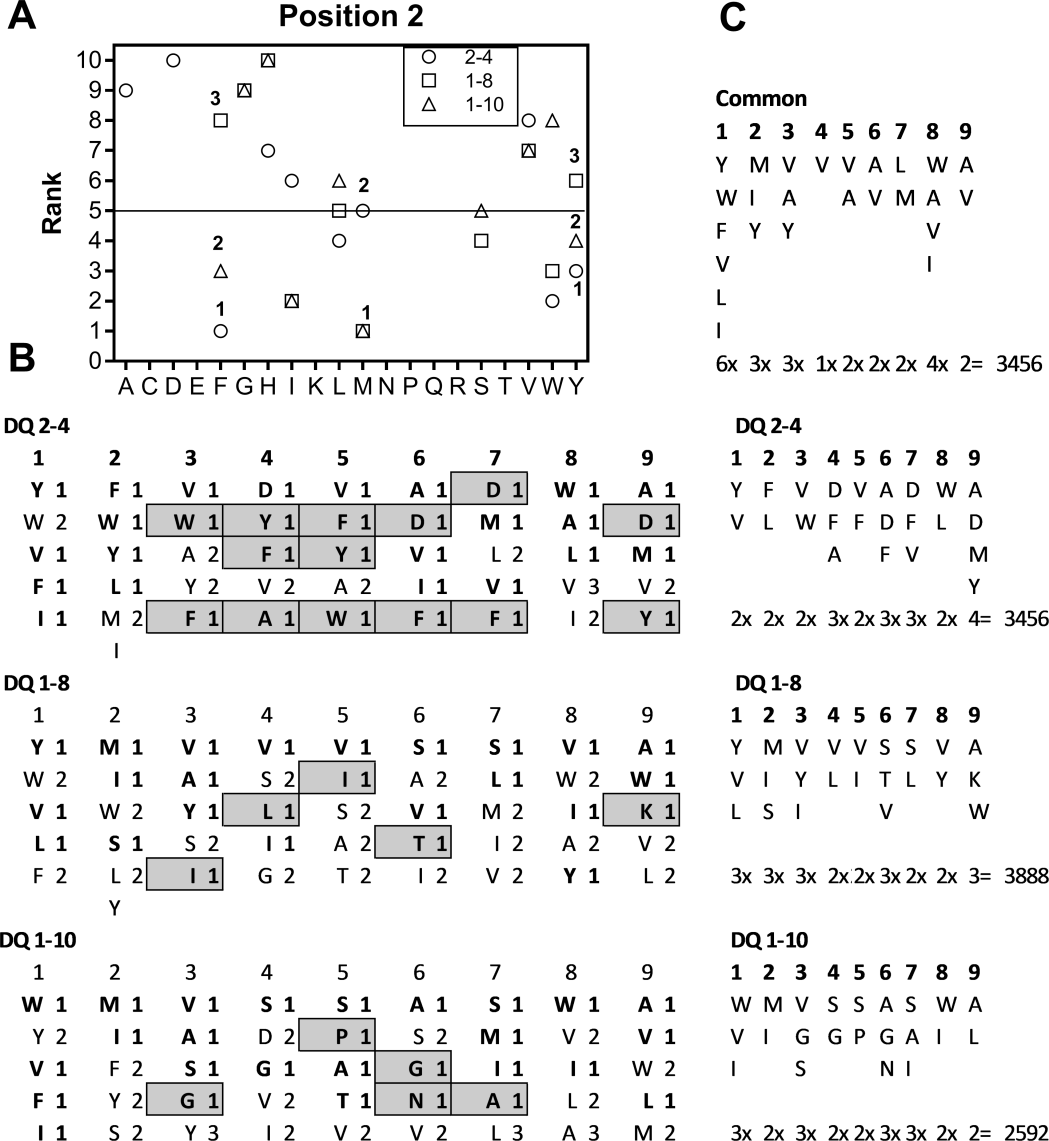
Selection of amino acids for combination sequences. (A) To determine the highest ranked amino acids selective for, or common to MPSLs for 3 IRL dolphin proteins (DQ1-08 □, DQ1-10 Δ and DQ2-04 ○), each of the 9 positions the ranking data for all 3 MPSLs were superimposed on a single graph (sample graph for position 2 is shown). (B). The top 5 amino acids at each position were given a value 1–3 depending on their rank in the composite graph For example, Tyrosine (Y) in position 2 would be assigned a value of 1 for DQ2-4, 2 in DQ1-10, and is not in the top 5 for DQ1-8; Methionine (M) would be given a value of 1 for both DQ1-8 and DQ1-10. (C) Combinations of the common or most selective amino acids amino acids used to generate sequences a total of 13,392 sequences were generated.

For example, scatter plots for the amino acid ranking at each of the 9 positions in the MPSLs derived from DQ2-4 and DQ1-10 are shown in (Fig 6). Amino acids that ranked highly only in DQ2-4, DQ1-10 or DQ 1–8, along with those amino acids that ranked highly in all 3 analyses, are described in the methods and presented in (Fig 7). A combinatorial arrangement of the amino acids from each of the nine positions that ranked in the top 5 and are common to all three analyses (i.e., active in all DQ proteins) generated 3,456 highly ranked 9-mer sequences from a theoretical 1,953,125 (i.e. 5^9^) possible sequences (see ‘common’ in Fig 7C). A search in the UniProtKB database (90,645,980 entries in release 2017–9 mined through PIR) for these 3,456 nonomer sequences identified 1,090 in proteins (31.5% see Table 4). This is a significantly higher hit rate (Х^2^ = 26,812, p<0.0001) than random expectations based on a similar search involving a list of 1,000 random 9-mer sequences that generated only 12 hits (a 1.2% hit rate). Hit rates were lower for sequences derived from specific DQ proteins; sequences from DQ 1–8 yielded 540 hits out of 3,888 (14%), DQ 1–10 yielded 223 hits out of 2,586 (9%); and DQ 2–4 yielded 178 hits out of 3,456 (5%) sequences. However, these hit rates were still significantly higher than the random peptide searches (Х^2^ = 454–5,274; p< 0.0001 for all 3). Notably, the sequences identified from the top 5 ranked amino acids common to both the model and physical combinatorial screen for the human DPA1*0103 and DPB1*0201 alleles was also significant, generating 50 hits out of 864 highly ranked peptides (a 5.8% hit rate; Х^2^ = 163, p<0.0001).

**Table 4:** Summary of hits from search of UniProtKB database (90,645,980 million entries).

	# combinations	# sequences In proteins		# sequences in list	
**Common**	3,456	1,090	32%	393	36%
**DQ1-8**	3,888	540	14%	129	24%
**DQ1-10**	2,586	223	9%	27	12%
**DQ2-4**	3,456	178	5%	29	16%
**Total**	**13,386**	**2,031**	**15%**	**578**	**28%**

Sequences identified in proteins listed in UniProtKB were further examined for relevance to dolphin health. In order to establish health relevance, we assembled a list of 55 known pathogens of marine mammals (some at genus level some at species level) of which 31 have been reported to occur in bottlenose dolphins [40–42] and assessed how many of these were identified among the organisms associated with the identified sequences. Sequences derived from aa common to all three haplotypes that were identified in proteins from the 55 known pathogens are listed in Table 4 (A summary of pathogen sources, for hits from the 3,456 sequences (‘common’ see above) and sample proteins identified are presented in the table). One third (393 of the 1,090) of the ‘common’ sequences identified were found to occur in microbes that are known to infect marine mammals, a significantly higher proportion than expected by chance (X^2^ = 295, p<0.0001) (similar tables summarizing source hits for sequences derived from the individual haplotypes are presented in S9–S11 Tables, and a complete table of each sequence identified with associated database IDs, protein and organism names is presented in S12 Table). A summary of the marine mammal associated microbes that were identified from sequences derived by our analysis is provided in Tables 5 and 6; 43 of the 55 genera were identified, 9 of the 11 genera reported in the Indian River Lagoon were identified, and 6 of these down to species level. While an average of 3,000 sequences per DQ protein were searched in the protein database, this only represents a minute fraction of the actual number of combinations for the top 5 ranked amino acids. A thorough exploration of the peptide space would require a search of 5^9^ = 1,953,125 sequences for each DQ protein. The search of sequences derived from the amino acids common to all 3 IRL haplotypes generated the most hits in terms of health relevance with 33 pathogen genera identified and 21 of those reported to occur in bottlenose dolphins. In contrast, a search performed using sequences derived from amino acids common to both screens of the human DP allele, and therefore not expected to have much connection to marine mammal pathogens, identified 3 of the genera found in marine mammals and only 2 of those reported to occur in bottlenose dolphins.

**Table 5:** Proteins and pathogens identified by MPSLA for dolphins from the IRL.

	Organism	#	a	Protein	#	a	Sequence			
1	*Absidia sp*.	1		Uncharacterized protein			VYAVAAMAA			
2	*Acinetobacter sp*.	9	+	Cell division protein FtsQ	5	+	FIVVVALVA	LIAVAVLAV	LYVVAVLIA	VIVVVVLVV
	* *			Competence protein ComE			FIVVVVLVA	LIAVVALVV	LYVVAVLVA	VIYVAALVA
	* *			Invasion protein expression up-regulator SirB			FYVVAALVV	LIVVVVLVA	VIAVAVMAV	VMAVVALVV
				LPS export ABC transporter periplasmic protein LptC			IIAVAALIA	LMVVVVLVA	VIVVAVLIV	YYAVVALIA
				Surface polysaccharide O-acyltransferase, integral membrane enzyme			IIVVVVLVA	LMVVVVMIA	VIVVVVLVA	
3	*Aeromonas sp*.	3	+	Sec-independent protein translocase protein TatB	2		VIAVVALVV			
				Tail length tape-measure protein			VMAVVALVA			
4	*Actinomyces sp*.	17	+	ABC transporter ATP-binding protein	6	+	FIAVAALAA	LIAVVVMAV	LIVVVVLIA	VIVVAALAV
	* *			ComEA protein			FIVVAVLAV	LIVVAALAA	LMAVVALVA	VIVVAALVA
	* *			Membrane protein, PF03706 family (Fragment)			FYVVAVLAV	LIVVAVLAV	LMAVVALVV	VIVVAVLVV
	* *			Permease, cytosine/purine, uracil, thiamine, allantoin family			IIVVVVLVA	LIVVAVLVV	VIAVAVLVV	VIYVAVMAA
				Putative stage III sporulation protein E			IMVVVVLVV	LIVVVALIA	VIAVVAMAA	VMAVAVLVA
				Signal recognition particle receptor FtsY			LIAVAALVV	LIVVVVLAA	VIAVVVLAA	VYAVVALAA
5	*Ajellomyces sp*.	1		Uncharacterized protein		+	LMAVVVLVV			
6	*Aspergillus sp*.	4		1,3-beta-glucanosyltransferase	5	+	FYVVAVLIV	VIAVAALVA	VIYVAVLVV	
	* *			Zinc finger protein klf1			IIVVVVLVV	VIVVVVLWA	WIAVAALAV	
				C2H2 finger domain-containing protein			LYVVAAMVA	VIYVAALVV	WIVVAVLAV	
7	*Bacillus sp*.	30	+	ABC transporter permease	12	+	FMAVAVMVV	IIVVVVMAA	LIVVAALVV	VIAVVALVA
				AI-2E family transporter			FYAVAALVV	IIYVVAMIA	LIYVVALAA	VIVVAVLVA
				Alkyl hydroperoxide reductase			FYAVAVLVV	IMAVAALVA	LMAVAVMVV	VMAVVVLIA
				Bacitracin transport permease bcrb			FYVVAALVV	IMAVAAMVA	LMAVVAMIV	VMAVVVLIV
				Cadmium efflux P-type ATPase			FYVVAVLAA	IMVVAALVA	LMVVAVLVA	VMAVVVMAV
				D-ribose ABC transporter substrate-binding protein			FYYVVALIV	LIAVAALAA	LMYVVVMAV	VMVVAVLIV
				DUF1453 domain-containing protein			IIAVAAMVV	LIAVAVLIA	LYAVAALVV	VMVVVAMVA
				Endoribonuclease			IIAVAVMVV	LIAVAVLVA	LYAVAVLVV	VYAVAVLAA
				Lipoprotein signal peptidase			IIVVAALVA	LIAVVVLVA	LYVVAALAV	
				Phage protein			IIVVAAMIV	LIAVVVLVV	VIAVVALIA	
8	*Bordetella sp*.	10	+	ABC transporter permease	5	+	FYAVAALWV	LMAVVALAA	VIAVVAMVA	WIAVAVLAA
				Flp pilus assembly protein CpaB			IIVVVALAA	LMVVAVLVV	VIVVAALVA	WIVVAVLIA
	* *			Receptor family ligand-binding protein			IMAVAALAA	LYAVAALAA	VMVVAALVA	
				Stress protection protein MarC			LIAVAAMAA	VIAVAALWV	VYAVAVLAA	
9	*Brucella sp*.	10	+	ABC transporter permease	1		VIVVAVLAV			
10	*Campylobacter sp*.	4	+	BAX inhibitor (BI)-1 like protein (UPF0005 domain)	3	+	IIAVAAMWA	VIVVVALIA
				GntP family permease			FIAVVVLIV	
				D-glycerate transporter (Predicted)			IMAVAVLIV	
				Uncharacterized protein			VIAVVVMIV	
11	*Citrobacter sp*.	1		Disulfide bond formation protein B	1		YMVVAVLVV			
12	*Clostridium sp*.	12	+	Uracil permease	6	+	FIAVVALAV	IMAVAALIV	LIVVAVLAV	VIVVVVMAV
	* *			Copper-exporting P-type ATPase A			FYAVAALIV	IMAVVVLVV	LIVVVAMIV	VIYVVVLIV
	* *			PTS system beta-glucoside-specific EIIBCA component			IIAVVALVV	IYAVAVLVV	LIVVVVLVV	WIAVAVLAA
	* *			Signal peptidase I			IIAVVVLAV	IYAVVAMIV	VIAVAALIA	WIVVVALVA
	* *			Sortase			IIAVVVLIV	IYVVVALIV	VIAVVVLAV	
	* *			Sporulation integral membrane protein YtvI			IIVVVALIV	LIAVAAMVV	VIVVAALAV	
							IIVVVAMVA	LIAVAVLVA	VIVVVALIA	
13	*Corynebacterium sp*.	36	+	Arabinosyltransferase C	14	+	FIVVAVLVA	IIVVVALVA	LIVVAVLIA	VIAVVAMAV
	* *			Beta-carotene 15,15'-monooxygenase			FIVVVALVA	IIVVVVMAV	LIVVAVLVA	VIAVVVLAV
	* *			Competence protein ComE-like protein			FMVVAALAA	IIYVAVMVA	LIVVVALAV	VIVVAVLVA
	* *			CvpA family protein			FMVVAVLIV	IMVVVVLAV	LIVVVALIV	VIVVVALVA
	* *			YceI-like domain protein			IIAVAALAA	IMYVVAMAV	LIVVVALVA	VIYVVALAA
	* *			CytoChrome c oxidase Caa3 assembly factor			IIAVAALAV	IYVVAALIA	LIVVVVLAV	VMAVAALAA
	* *			Major facilitator transporter			IIAVVALIA	LIAVAALAA	LIVVVVLVV	VYAVAALWA
	* *			Septum formation			IIAVVVLIV	LIAVAALAV	LMAVVVLAA	WIAVVALVA
				Serine protease			IIAVVVLVA	LIAVVALVA	LYAVAALAV	WIAVVVLAV
				Signal peptidase I			IIAVVVLVV	LIAVVVLAV	VIAVAALIV	WMAVAALAV
				Sodium/hydrogen exchanger			IIVVAVLAV	LIAVVVLIA	VIAVAVLVA	WMAVAVLVA
				Phage tail tape measure protein, TP901 family (Fragment)			IIVVAVLWA	LIAVVVLIV	VIAVAVMAV	WYAVVALAV
				Phosphatidate cytidylyltransferase			IIVVAVMVA	LIVVAVLAA	VIAVVALAV	YIVVAVLVA
				Polyisoprenoid-binding protein YceI			IIVVVALIA	LIVVAVLAV	VIAVVALIV	
14	*Edwardsiella sp*.	4		Lipoprotein signal peptidase			WIVVAVLIV			
15	*Enterobacter sp*.	5	+	Inner membrane peptidase. Serine peptidase. MEROPS family S49	6	+	FYAVVALVA	IIVVAVLIV	LYVVVALVV	
	* *			Major facilitator superfamily MFS_1			FYAVVALVV	IYAVVALAA	VIAVVVMAA	
	* *			Methyl-accepting chemotaxis sensory transducer TarH			FYVVVALVV	LIVVAVLIA	VIVVVALAA	
	* *			Probable lipid II flippase MurJ			FYVVVAMVV	LIVVVALAA	VIVVVVLAA	
16	*Enterococcus sp*.	7	+	Multidrug ABC transporter permease	2	+	FIVVVALIA	LIVVAALIV	LMVVAALIV	
				Alpha/beta hydrolase			IMVVAALIA	LIVVAVLAA	LMVVVALIV	
							LIAVVVLIV	LIVVVALVV	VYYVVALIV	
17	*Escherichia sp*.	3		Glutathione-regulated potassium-efflux system protein (K(+)/H(+)antiporter)	4	+	FIAVAALAA	LYVVAALVV	LYVVVALVV	VIAVAVMWV
				Hydrogenase-4 component B / Formate hydrogenlyase subunit 3			VIAVVVLVA	VIAVVVMAA	VIVVVALAV	
18	*Fusarium sp*.	2		Amino acid transporter	2		VIVVAALAV			
	* *			Glutamyl-tRNA amidotransferase subunit A			LMVVAALAV			
19	*Influenza A virus*	1		Hemagglutinin	1		IIVVAVLAA			
20	*Helicobacter sp*.	1	+	Proline and betaine transporter	2	+	IIAVVALIV	YIVVVALIA		
21	*Kingella sp*.	1		Uncharacterized protein		+	IIAVAVLAV			
22	*Klebsiella sp*.	3		Lipoprotein releasing system transmembrane LolC	3	+	IIAVAALVA	IIAVVAMAV	LYVVAALVV	
23	*Micrococcus sp*.	3	+	ATP-binding cassette, subfamily B	3	+	LMVVAALIV	IIAVVAMVV		
	* *			Predicted arginine uptake transporter			WIAVAVMAV			
24	*Moraxella sp*.	3		Mechanosensitive ion channel protein MscS	2	+	LMAVVVMAA	VYVVVVMVV		
	* *			Cytochrome c oxidase accessory protein CcoG			LIVVAAMIA			
25	*Morganella sp*.	2		Iron ABC transporter permease	3		LIAVVALIA	LYVVAALVV		
26	*Mortierella sp*.	1		Uncharacterized protein		+	LIVVAVMIA	IIVVAVLVV		
27	*Mycobacterium sp*.	132	+	ABC transporter permease	61	+	157	LIVVVVLAV	VIVVAVLIA	LIAVAVLAV
	* *			Acyl-CoA dehydrogenase			FIAVAVLAV	LIVVVVLIA	VIVVAVLIV	LIAVAVMIA
	* *			Adenosylcobinamide-GDP ribazoletransferase			FIAVVALAA	LIYVVAMIV	VIVVVALAA	LIAVVALAA
	* *			Arsenic transporter			FIVVAVLIV	LMAVAVLAA	VIVVVALIV	LIAVVALAV
	* *			Cadmium-translocating P-type ATPase			FIVVVVLAA	LMAVVALAA	VIVVVVLAA	LIAVVALIV
	* *			ComE operon protein 1			FIVVVVLAV	LMAVVAMAA	VIVVVVLAV	LIAVVVLAV
	* *			Cytochrome C-type biogenesis protein ccdA			FMAVAVLAV	LMVVAALAA	VIVVVVLIV	LIVVAALAA
	* *			Dipeptide-binding protein DppE precursor			IIAVAVLIV	LYAVAALAA	VMAVAVLAA	LIVVAALAV
	* *			Ethanolamine permease			IIAVVALIA	LYAVAALAV	VMAVAVLAV	LIVVAALIV
	* *			Exopolyphosphatase			IIAVVVMIV	LYAVAVLAA	VMAVVALAA	VIAVVAMAA
	* *			Flotillin			IIVVAALIA	LYVVAALWA	VMVVAALAV	VIAVVAMAA
	* *			Haloacid dehalogenase			IIVVVALAA	VIAVAALAA	VIAVVVLAA	VMVVAAMWV
	* *			Long-chain-acyl-CoA dehydrogenase			IIVVVVLAA	VIAVAALAV	VYAVAALAA	VIAVVVLAV
	* *			MCE-family protein MCE1A (Fragment)			IIVVVVLAV	VIAVAALIA	VYAVAALWA	VIAVVVMAA
	* *			Modulator of FtsH protease HflK			IMAVAALAA	VIAVAALIV	VYAVVALAA	VIAVVVMAV
	* *			Murein biosynthesis integral membrane MurJ			IMAVAALIA	VIAVAAMIA	VYAVVALAV	VIAVVVMIA
	* *			Oxidoreductase molybdopterin-binding protein			IMVVAALAV	VIAVAVLAV	VYAVVVMAA	VIAVVVMIV
	* *			Protein-export membrane protein SecF			IYVVAALAA	VIAVAVLIA	VYVVAALIA	VIVVAALAV
	* *			Thioredoxin			LIAVAALAA	VIAVVALAA	WIAVAALIA	WMVVAVLAA
	* *			Type VII secretion integral membrane protein EccD			LIAVAALAV	VIAVVALAV	WIAVAAMAV	YIAVAAMAA
	* *			UDP-phosphate galactose phosphotransferase			LIAVAALIA	VIAVVALIA	WIAVAVLAA	YMAVAALAA
	* *			Virulence factor Mce			LIAVAVLAA	VIAVVALWV	WIAVVALAA	YYAVVVLAA
28	*Nocardia sp*.	10	+	Cytochrome C oxidase assembly factor CtaG-related	5	+	FIAVAALIV	IIVVAAMVA	LMAVAALVV	VIVVVVLAA
	* *			HTH-type transcriptional repressor			FIVVAAMVV	LIAVAALAV	LYAVAALAA	VIVVVVLVA
	* *			MFS transporter			FYAVVVLIV	LIAVVALAV	VIAVAALVA	VIVVVVLVV
	* *			Thioredoxin			IIAVAALAA	LIAVVALVV	VIAVVALIA	VYAVAALAA
				Type VII secretion integral membrane protein EccD-like protein			IIAVAVLVA	LIVVAVLAA	VIAVVVLAA	WIVVAVLAA
							IIAVVALVV	LIVVVALIV	VIVVVALIV	
29	*Photobacterium sp*.	5	+	Macrolide export ATP-binding/permease protein MacB	2	+	FIAVVVLIV	FMAVAVMAA	LIAVVVLVA	
	* *			Electron transport complex subunit B			VIVVVALIV	VIAVAVLAA		
30	*Providencia sp*.	5		NADH-ubiquinone/plastoquinone complex I subunit	3	+	FIVVVVLIV	LIAVAVLVA	LIAVVALVA	LIAVVVLVA
	* *			LemA family protein			LIAVVVMAA	LYVVAALIV	LYVVAALVV	
31	*Pseudomonas sp*.	61	+	Acyltransferase family protein	15	+	61	LIAVVVLIA	LMAVVALIA	VMVVVALAV
	* *			Allantoin permease			FYAVVALAV	LIAVVVMAV	LMVVAVMAA	VMVVVVMWA
	* *			Arabinose efflux permease family protein			IIVVAVMAA	LIVVAALAA	LMVVVVLIA	VYVVAALIV
	* *			TrbK entry exclusion protein			IIVVVALAA	LIVVAALAV	LMVVVVMAA	VYVVAVLAV
				Chemotaxis sensory transducer			IMVVVALAV	LMVVVVMWA	LIVVAVLAA	WIAVAALIV
	* *			Cytochrome o ubiquinol oxidase subunit IV			LIAVAALAA	LIVVAVLAV	LYAVAALIV	WIAVAVMIV
	* *			Deoxyribonuclease			LIAVAALAV	LIVVVALAA	VIAVAVMAA	WIVVAAMIV
	* *			TspO and MBR related proteins			LIAVAVLAV	LIVVVVMAV	VIVVAAMIA	WIVVAVMIV
	* *			Endolytic murein transglycosylase			LIAVVALIA	LMAVAAMAV	VIVVAVMAV	YIVVAVLAA
	* *			Heat-shock protein			LIAVVAMAV	LMAVAVLAV	VMVVAALIA	LMAVAVLIV
32	*Rhodococcus sp*.	17	+	ABC transporter permease	10	+	FIAVVALAA	LIAVAVLAV	LIVVVVLVA	VIAVVALAV
	* *			Arabinosyltransferase			FIVVVALIA	LIAVVALIA	LMAVAVLIV	VIVVAVLVV
	* *			Cell wall arabinan synthesis protein			FYVVVVLVA	LIAVVVLAV	LMVVAALAA	VYAVVALVA
				FMN-binding glutamate synthase family protein			IIVVVALVV	LIAVVVLVV	LMVVAVLAV	WIAVAALAV
				Histidine kinase			LIAVAALAV	LIAVVVMVV	VIAVAALAA	WIVVAVLVA
				Methylamine utilization protein MauD			LIAVAALIV	LIVVAALVA	VIAVAALIV	WIVVAVLVV
				NADH-Ubiquinone/plastoquinone (Complex I), various chains family protein			LIAVAALVA	LIVVAALVV	VIAVAALVV	WIVVVVLVA
				Pilus assembly protein TadE			LIAVAALVV	LIVVAVLVA	VIAVAAMVV	WMAVVVLIA
				Sensor histidine kinase DcuS			LIAVAVLAA	LIVVAVLVV	VIAVVALAA	YIAVAALVV
33	*Rhizopus sp*.	1		Uncharacterized protein		+	IIVVVVMVV			
34	*Serratia sp*.	10	+	Macrolide export protein MacA	4	+	FIAVVALIA	IIAVAAMAV	IIAVVAMAV	LIVVVALAA
	* *			D-galactonate transporter			LIVVVVLVA	VIAVAAMAV	WIVVAVMAA	
35	*Sporothrix sp*.	2		Autophagy protein	1	+	LIAVVVLVV	VIVVAVLWV		
36	*Staphylococcus sp*.	3		Multidrug MFS transporter	2	+	IIAVAALIV	IYAVVALVV	LIVVAVLIA	LYVVVALIV
37	*Streptococcus sp*.	8	+	Major facilitator transporter	4	+	FYAVAALVV	LIAVVALIV	LMVVAALIV	YIVVVVLVA
	* *			PTS system beta-glucoside-specific IIA Glc family			IIAVVVLIV	LIVVVVLVA	VIAVVVLAV	
				Septation ring formation regulator EzrA			IIVVVALAA	LIVVVVLVV	VIYVAVLIA	
				Phosphate transport system permease protein PstA			IYVVVALIA	LMAVAVLAV	WIAVAVLAA	
38	*Vibrio sp*.	16	+	Acriflavin resistance protein	6	+	FIVVVVLVV	LMVVAALIA		
	* *			DeoR faimly transcriptional regulator			FMVVVVLAV	VIAVVVLIA		
	* *			Dipeptide and tripeptide permease A			IIAVVALAA	VMVVVALAA		
	* *			Flagellar basal body-associated protein FliL			IIVVAALIV	VMVVVALIA		
	* *			Homoserine/homoserine lactone efflux protein			LIAVVALVV	WIAVAVLAV		
	* *			Thiol-disulfide isomerase			LIAVVVLIV			
	* *			Putative ABC transporter, permease component			LIVVAVLWV			

The 3,456 sequences derived from amino acids common to the 3 Model Positional scanning libraries for (DQ1-8, DQ1-10 and DQ2-4) were searched for protein matches in the UniProtKB database through the Protein Information Resource (PIR). The search generated 1090 matches with 393 sequences identified in proteins of microbes associated with marine mammals. Sequence matches for proteins originating from reported pathogens in marine mammals are summarized here. Columns listed as (#) refer to numbers identified, or (a) list includes undefined species or proteins. Full details are supplied in S12 Table.

**Table 6:** Marine pathogens identified from sequences derived from MPSLs for dolphins from the IRL.

	Org.*	Genus		Reported species identified		Org.*	Genus		Reported species identified
1	IRL	Aeromonas sp.	✓		28	Cet	Absidia sp.	✓	
2	IRL	Bacillus sp.	✓		29	Cet	Acinetobacter sp.	✓	
3	IRL	Campylobacter sp.	✓		30	Pin	Corynebacterium sp.	✓	*Corynebacterium phocae*
4	IRL	Candida. sp.	✓	*Candida albicans*	31	Cet	Fusarium sp.	✓	
5	IRL	Clostridium sp.	✓		32	Pin	Bordetella sp.	✓	*Bordetella bronchiseptica*
6	IRL	Edwardsiella sp.	✓	*Edwardsiella tarda*	33	Cet	Citrobacter sp.	✓	*Citrobacter freundii*
7	IRL	Enterobacter sp.	✓	*Enterobacter cloacae*	34	Cet	Influenza A	✓	
8	IRL	Escherichia sp.	✓	*Escherichia coli*	35	Cet	Kingella sp.	✓	* *
9	IRL	Helicobacter sp.	✓	*Helicobacter pylori*	36	Pin	Leptospira sp.	✓	*Leptospira interrogans*
10	IRL	Klebsiella sp.	✓	*Klebsiella pneumoniae*	37	Cet	Micrococcus sp.	✓	
11	IRL	Plesiomonas sp.			38	Pin	Mycoplasma sp.	✓	
12	IRL	Pseudomonas sp.	✓	*Pseudomonas aeruginosa*	39	Cet	Moraxella sp.	✓	
13	BD	Actinomyces sp.	✓	*Actinomyces viscosus*	40	Cet	Mortierella sp.	✓	
14	BD	Ajellomyces sp.	✓	*Ajellomyces dermatitidis*	41	Pin	Rhodococcus sp.	✓	*Rhodococcus equi*
15	BD	Aspergillus sp.	✓	*Aspergillus niger*	42	Cet	Rhizopus sp.	✓	
16	BD	Brucella sp.	✓	*Brucella ceti*	43	Cet	Serratia sp.	✓	*Serratia marcescens*
17	BD	Enterococcus sp.	✓	*Enteroccocus faecalis*	44	Cet	Sporothrix sp.	✓	*Sporothrix schenckii*
18	BD	Morganella sp.	✓		45	BD	Blastomyces Sp.		
19	BD	Mycobacterium sp.	✓	*Mycobacterium tuberculosis*	46	BD	Coccidioides sp.		
20	BD	Nocardia sp.	✓	*Nocardia brasiliensis*	47	BD	Trycophyton sp.		
21	BD	Photobacterium sp.	✓	*Photobacterium damselae*	48	BD	Lacazia sp.		
22	BD	Proteus sp.	✓	*Proteus mirabilis*	49	Pin	Dermatophilus sp.		
23	BD	Providencia sp.	✓	* *	50	Cet	Pasteurella sp.		
24	BD	Salmonella sp.	✓		51	Cet	Abiotrophia sp.		
25	BD	Streptococcus sp.	✓	* *	52	Cet	Actinobacillus sp.		
26	BD	Staphylococcus sp.	✓	*Staphylococcus epidermidis*	53	Cet	Cetobacterium sp.		
27	BD	Vibrio sp.	✓	*Vibrio parahaemolyticus*	54	Cet	Mucor sp.		
					55	Pin	Bisgaardia sp.		

*Reported in bottlenose dolphin in Indian River Lagoon IRL, other bottlenose dolphin, BD; other cetacean, Cet; or pinniped, Pin.

Sequences derived from Model Positional scanning libraries for 3 dolphin proteins (DQ1-8, DQ1-10 and DQ2-4) were searched for protein matches in the UniProtKB database through Protein Information Resource (PIR). The bacterial, fungal or viral sources of the matching proteins that have been reported to infect marine mammals are summarized.

Perusal of the data obtained from the protein database revealed a wealth of information: of the four combined searches the sequence that occurred most frequently in different organisms was VIVSSGAIA. It was identified in 161 different species, in 5 genera, and is located in the protein Glutamate-5 kinase. The most widely targeted group of proteins was the ABC transporter family, with 106 sequence hits identified, certain members of which are known to be associated with bacterial virulence (see [43] for review). The most widely identified set of organisms was the genus Mycobacterium, with 188 sequences recognized, of which the most recognized species was *M*. *abssessus* which had 22 sequences associated with it. There were 294 sequences identified in uncharacterized proteins. The sequence identified in the widest range of proteins in the database was LIAVAVLAV, which occurs in at least 8 different proteins including; Serine-threonine protein kinase, ABC transporter/permease, Chemoreceptor McpA, Glutaredoxin, Hflk protein, Secretion protein EccD and Flagellar L-ring protein precursor. Interestingly, an analogous sequence VIVVAVLAV was identified in ABC transporter/permease in *Brucella ceti*, a pathogen of interest for dolphins in Florida.

The search for sequences derived from the analysis for aa common to the 3 haplotypes yielded hits in 38 genera; the most frequently represented was *Mycobacterium* with 132 species identified from 157 sequences. There were 212 sequences associated with uncharacterized proteins, and sequences were most frequently identified in transporter proteins. Searches derived from the analysis for individual DQ haplotypes also revealed interesting results: analysis of data for DQ1-8 identified the greatest number with relevance to marine pathogens sequences (129), more than DQ2-4 (29) or DQ1-10 (27). Sequences selective for DQ1-8 represented in the protein database contained 62 sequences that occurred in uncharacterized proteins and over 40 in the ABC transporters. Again, the most frequent genus identified was *Mycobacterium* with 26 sequences in 51 species. This search identified 5 genera not found in the “common” search (*Bordetella*, *Proteus*, *Salmonella*, *Leptospira* and *Sporothrix*) and 2 genera identified were specific to this DQ haplotype; *Proteus sp*. (LIILISLYK), and *Salmonella s*p. (VMILVVLVW). The search for haplotype DQ1-10 identified 2 genera not identified in the “common” search and 1 genera specific to this haplotype; *Candida* sp. (VIVSSGAIA and IIVSSGAIA). The genus with the most sequences identified for DQ1-8 was *Pseudomonas* (7) and the most frequent protein identified in this search was Glutamate-5 Kinase. The search for haplotype DQ2-4 generated a high proportion of sequences in uncharacterized proteins (15); of the sequences associated with proteins, most were located in ABC transporters. The genus with the most sequences (5) was *Mycobacterium*, sequences were identified in 9 species; and the second most frequent genus *Nocardia* was identified by 3 sequences that occurred in 4 species. There were no genera unique to this search however the dolphin related fungus *Ajellomyces dermatitidis* was identified by the sequence YFWFFAVLA (in the protein ATPase). While a similar organism *Ajellomyces capsulatus* was identified in the DQ1-8 related search, it was recognized by a completely different sequence (VIVVVSSVA) in a different protein (Hydroxyacyl-Coenzyme A dehydrogenase type II).

There were 210 sequences that were identified proteins from the 13 pathogens of concern for dolphins in the IRL [42,44]. Four were identified in *Vibrio parahaemolyticus*; VMVSSGAIA, WIGGSAIIL, IISGSGIIA and VIYLISLVA, the first two sequences were identified in the proteins Glutamate 5-kinase and Maltose O-acetyltransferase, respectively, and the latter two sequences were located in uncharacterized proteins. Two sequences FIAVAALAA and VIAVAVMWV were identified for *Escherichia coli*. Two sequences were also identified for *Mycobacterium marinum* (VIVVIVLVA VIVSSGAIA). Eight sequences were identified for *Mycobacterium tuberculosis*, indeed all four searches identified sequences for this organism. *Edwardsiella tarda* was identified in 2 searches (common and for DQ1-10), and it was also identified in microbiological cultures from 5 of 8 dolphins tested from the IRL. Of the two most frequent genera in the microbiological cultures from the IRL (found in 6 and 7 of the 8 dolphins respectively) sequences for *Aeromonas* sp. were identified but no sequences were found for *Plesiomonas*,. Sequences for 9 of the 10 genera identified in the microbiological cultures however were identified. Lobomycosis is known to be a problem in bottlenose dolphins along the Florida Atlantic coast and the IRL [45–49] but the infectious agent *Lacazia (Loboa) loboi* was not identified among any of our searches of peptide sequences. However, at the time of submission there were only 21 entries and 5 reported proteins in the PIR database for this organism.

**Fig 8:**
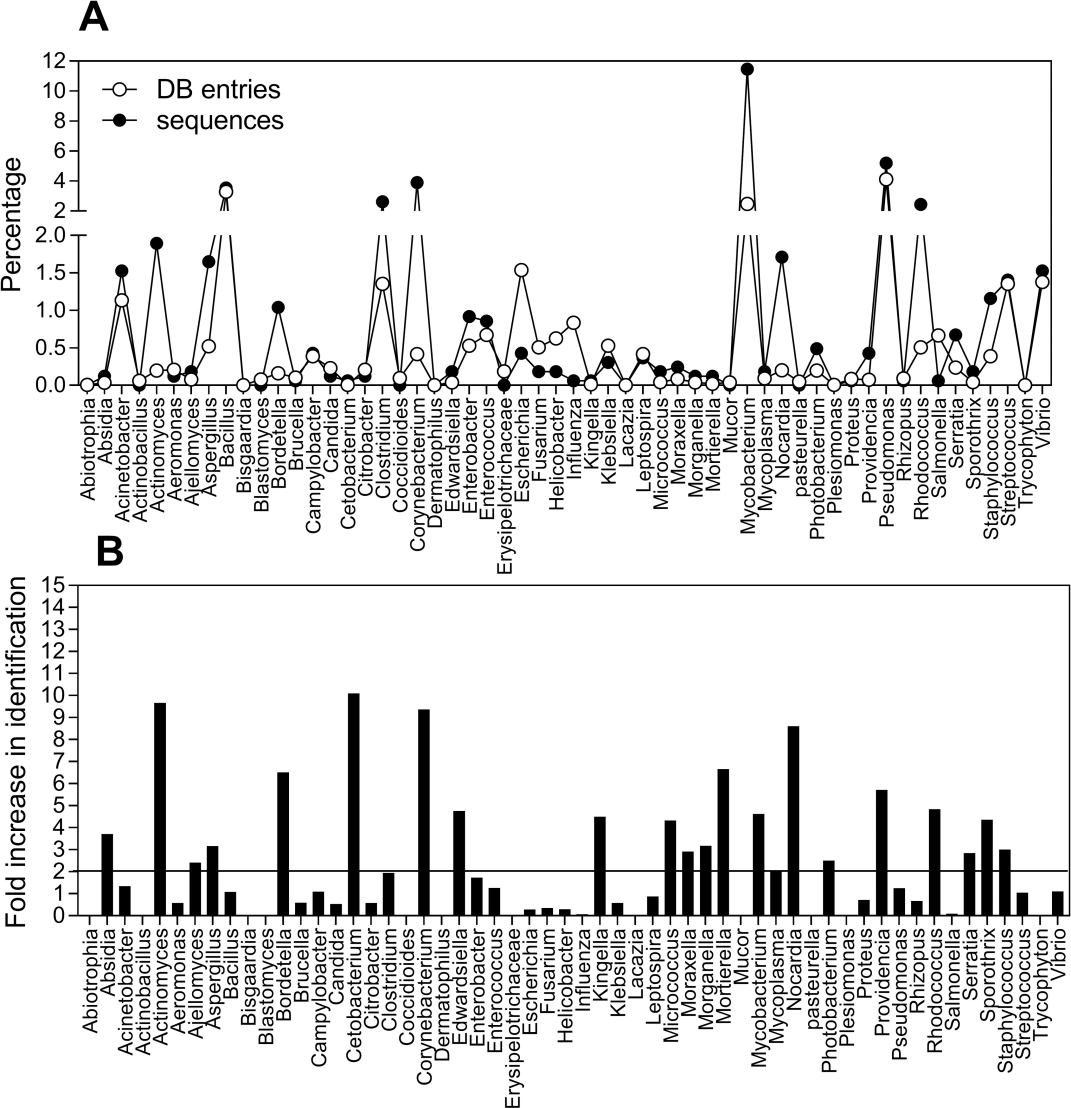
Relative occurrence of genera in UniProt database and in current search for marine related pathogens. A. Percentage of entries for named genera in the database compared to the percentage of sequences identified in named genera derived from MPSLA. B. Fold increase in identification of named genera.

We analyzed the results obtained from the protein database for confidence; survey of the 90 million entries revealed heavily skewed numbers of entries for certain organisms, in fact just five genera (*Escherichia*, *Pseudomonas*, *Mycobacterium*, *Bacillus and Clostridium)* account for more than 10% of all entries. To ascertain the veracity of our searches, we identified the number of entries in the database for each of the 55 marine related genera and expressed values as a percentage of total number of entries. We also determined the number of sequences identified for each genera and expressed this as a percentage of the total number of sequences identified (Fig 8A). Increases above 2 fold (sequences/entries) were taken as evidence that the search identified the genera beyond expectations for a random sampling. Twenty-one genera were identified as having at least a 2 fold or greater hit rate by our search (Fig 8B). This is exemplified by *Mycobacterium* that had a 4,6 fold higher hit rate than expected although this genus is one of the more frequent entries in the database. It is interesting to note that for several genera a much lower hit rate than would be randomly expected was observed, for example for *Escherichia sp*. had 3 fold fewer sequences identified than would be expected randomly. This may be evidence that different MHC proteins are directed against specific species.

### Discussion

The recent availability of genetic data for MHC class II alleles present in estuarine and coastal populations of bottlenose dolphins in Florida gave rise to the question of differential fitness between populations [50]. In the present study we wished to explore whether the reduction in allelic abundance in MHC II DQ found in dolphins of the Indian River Lagoon (IRL) as compared to their Atlantic neighbors (Table 1) was reflected by a reduction in immunocompetence. We wanted to determine whether diminished repertoires of peptides were recognized by these MHC proteins or whether the DQ alleles that dominated in the IRL population resulted in DQ proteins that were generalists and thereby compensated for the loss of other alleles in this population. To begin to address this issue, we needed to determine and compare the affinities of peptides that bound to the different DQ proteins arising from the combined DQA and DQB alleles of interest. Our initial approach analyzed the affinities of a series of peptide sequences as predicted by the MHC II binding algorithm *NNAlign*. This algorithm allows for user input of the MHC II alpha and beta protein sequences which enabled us to predict the binding affinities using the same list of 7,647 amino acids for each of four DQ proteins derived from alleles occurring in the IRL, and to compare the predicted affinities to those derived from a different dolphin DQ protein and from other cetacean species. The predicted affinities found for protein sequences derived from DQ alleles in the IRL were weaker than affinities for a predicted dolphin protein downloaded from Genbank, however, the predicted affinities observed were not very different to those observed for DQ proteins from other cetaceans, e.g. sperm whale. Significant overlap was observed for the sequences that were designated as active (binding affinity <10,000nM) between DQ proteins in the IRL. Only 534 of 7,406 sequences were selective for an individual DQ protein (532 sequences were predicated to bind only to DQ 1–8 and only 2 were unique to DQ 2–4), suggesting that these proteins may indeed be generalists. Unfortunately this approach provided little insight into the repertoires of peptides that bind to the individual DQ proteins and such inferences could not be confirmed.

**Fig 9:**
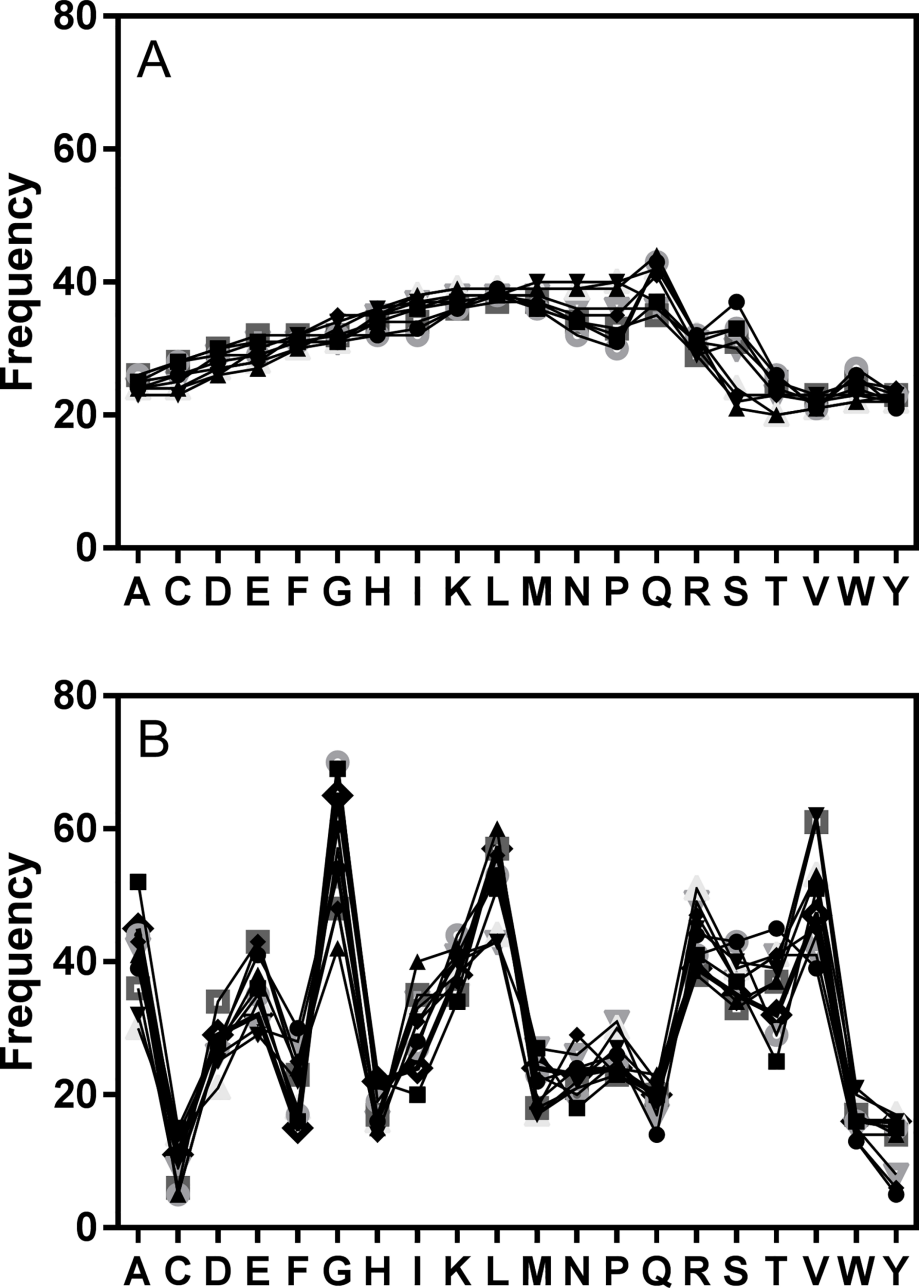
Frequency of amino acids at each position of a decamer peptide sequence. The frequency of each of the 20 amino acids (single letter code) was determined for each of the 10 positions of the peptide (one symbol for each position) from (A) a list of 616 sequences designed to have near equal distribution or (B) a list of 616 peptides derived from viral proteins.

Our second approach, however, harnessed the power of mixture based combinatorial libraries to provide such insight. The essence of a combinatorial library is equal representation of each amino acid at each position of a peptide length and the possible numbers of combinations can be in the trillions. Thereby, combinatorial libraries can readily encompass the complete repertoire of peptides that will bind to a particular MHCII protein. We used the binding affinities of 7,364 nonamer sequences to retroactively generate the binding affinities of the mixtures in a positional scanning combinatorial library. This set of calculated affinities represents the data obtained on screening the Positional scanning library in a traditional binding assay. From the four searches performed for the DQ proteins, 38 of the 53 known marine mammal pathogens and 18 of the 24 bacterial or fungal agents reported in bottlenose dolphins were identified, and 12 of those were identified down to species level. The advantage of modelling a combinatorial library is that it can be made available to entities that lack the physical and financial resources to conduct the physical experiments. Naturally any physical experiment will have more dependable results, but this approach of a modeled analysis using the binding algorithms and protein database searches will only improve with time as each of the components is refined. Indeed many of the assumptions made in this study will need further refinement. The amino acid list fed to the MHC II algorithm has small disparities in amino acid frequency (up to 2 fold differences, Fig 9A), this was much lower than frequencies found for a natural sample of peptides (>10 fold differences, Fig 9B) and while considered unlikely, it is possible that such differences were sufficient to influence rankings generated by the model library. Also in future studies it would also be useful to include amino acid rankings derived using algorithms other than *NNAlign*,which may shed light on bias inherent to the current system. In this initial study only a tiny fraction of the possible combinations of the top 5 ranked amino acids were combined and searched as sequences in the protein database, indeed even the choice of 5 amino acids as a cutoff was arbitrary and would need further evaluation. The search in the protein database for sequences derived from DQ1-8 yielded few results, suggesting that connectivity was not established with the amino acid choices and a greater number of combinations may need to be searched for a fuller evaluation.

In spite of the limitations mentioned, above, this initial study has demonstrated a method for bridging the genetic data (variance observed in MHCII alleles) through the protein level (prediction peptides recognized by the MHC protein variants) to the target (identification of pathogens). We were able to demonstrate strong correlations for the amino acids recognized by these proteins, and that the amino acids predicted to rank as most active did indeed generate sequences that not only occurred in proteins but in proteins from species known to infect this population of marine mammals. The most abundant proteins and organisms were also organisms related to infection in marine mammals, thus use of the filter of marine causing diseases, though useful, was not critical. High success for identifying proteins in pathogens for marine mammals was found when amino acids chosen were common to the DQ proteins. This suggests these DQ proteins may indeed be generalists, but sequences unique to DQ1-10, DQ2-4 and DQ1-8 were also identified, indicating these haplotypes may also function in specific pathogen recognition. Evidently, a more in depth study is required now that we have demonstrated the means. The data obtained from these analyses would benefit to research scientists, wildlife managers and policy makers through enhancing the specificity of population health studies and providing detailed susceptibility concerns. Rather than attempting to speculate on a population’s vulnerability to a disease outbreak based solely on genetic diversity, a disease threat can be explicitly probed against haplotypic binding potential of the dominant MHC allelic makeup. Even populations with high allelic diversity can be at risk from pathogens to which they are unable to recognize. Once the data has been corroborated by physical experiments, results would be of great value to databases like the Marine Mammal Health Monitoring and Analysis Platform (MMHMAP) [51]. The potential for MPSLA is significant, now rather than being limited to performing binding studies using peptide sequences derived from a pre-established target protein (for most MHCII proteins established targets are relatively few), target proteins can be identified through MPCL analysis and binding peptides can be evaluated physically once their role in a pathogen has been confirmed. Thus this methodology will be much more attainable for a variety of species, including non-model organisms and populations for which parasite load data is not available. Genetic variation in MHC alleles (MPSLA can be applied to both MHC class I and II) in distinct populations can now be linked to alterations in binding repertoires of expressed MHC proteins and used in the identification of target pathogens. Ultimately this approach may be used by researchers to evaluate risk in outbreaks of emerging diseases.

## Methods

### Peptide binding affinities for DQ alleles

To determine the peptide sequences likely to be recognized by unique MHC DQ heterodimeric proteins, we generated a large number of peptide sequences. These short peptides were then analyzed in conjunction with cetacean DQ receptor proteins to predict peptide binding affinities for different DQ molecules. This was achieved by first preparing a long sequence (7,647) of amino acids (aa) designed to have near to equal representation of each of the 20 L-amino acids (S1 Fig). This sequence was then supplied to *NN-Align* through the server (NetMHCIIpan 3.1 [30]) in combination with sequences for DQA and DQB derived from different alleles from cetacean species. The algorithm predicted binding affinities for 13-mer peptides (n = 7,634 drawn from the 7,647 aa sequence) a length typically bound by MHC receptor proteins. DQ protein sequences of four species from three cetacean families (Delphinidae n = 2, Phocoenidae n = 1 and Physeteridae n = 1) were downloaded from Genbank and used as standards for predicting binding affinities across the order; accession numbers were as follows: 1) Bottlenose dolphin *Tursiops truncatus* (Standard) DQA: XP_004317963.2,DQB: ABS58529.1; 2) Killer whale *Orcinus orca*: DQA:XP_004285666.1, DQB*0101: XP_012394439.1; 3) Finless porpoise *Neophocaena phocaenoides*: DQA: ALB25544.1, DQB: ALB25548.1; and 4) Sperm whale *Physeter macrocephalus*: DQA XP_007123886.1 DQB: XP_007123885.1. Most of these protein sequences were predicted from whole genome sequencing projects (WGS). Because a WGS generates a single consensus sequence from a diploid animal, these may not represent a true wildtype either individually as DQA or DQB alleles or together as a DQA/DQB haplotype. We therefore also used experimental data from Florida bottlenose dolphin populations recently documented in a companion paper by Pagán *et al*. Sequencing data from this study was limited to the DQA and DQB peptide binding region (i.e., exon 2), thus PBR sequences were inserted into the full length DQ protein sequence for standard dolphin described above (S2 Fig) and will be referred to as derived DQ proteins. Including the standards detailed above, a total of three DQA and four DQB PBR alleles were examined from dolphins. All except the DQB standard were found in both estuarine and Atlantic dolphins (Table 1). Three of the DQA and DQB PBR allelic combinations were found in homozogyotes from estuarine populations (Indian River Lagoon, IRL), and thus represent a confirmed haplotype. Exon 2 in *DQA* and *DQB* correspond to the amino acids located in the binding pockets of the expressed protein and it is acknowledged that mutations in this region have the greatest effect on peptide binding affinity [5]. Information submitted to the server (NetMHCIIpan 3.1) was as follows: A single query sequence of 7,647residues in FASTA format, Peptide length = 13, Threshold for strong binder (% Rank) = 2, Threshold for weak binder (% Rank) = 10; full length DQ Alpha and Beta chain protein sequences were individually uploaded. The affinities of active 13-mer peptide sequences for eight DQ proteins were compared and plotted (GraphPad Prism 6.1 software). To determine sequence overlap for Fig 3, only sequences with predicted affinities below 10,000 nM (designated as active binding peptides) were included in the analysis all 4 lists generated for (DQ 1–1, 10 = -8, 1–10 and 2–4) were combined and any duplicate sequences removed. the remaining sequences were then identified as occurring in one, two, three or all four peptide lists.

### Model positional scanning library analysis (MPSLA)

To model a positional scanning library, we generated hypothetical mixtures using the nonamer core of the 7,634 13-mer sequences generated by *NNAlign* (NetMHCIIpan 3.1). For example, to calculate the activity of a hypothetical mixture with the following nonamer core: AXXXXXXXX all sequences with alanine (A) at position 1 and their corresponding affinities were extracted from the list generated by *NNAlign*. Binding activity of the mixtures was then calculated by employing the harmonic mean [36] which is dominated by the minimum of its arguments; the equation is; H = 1 / (∑_(i = 1)_^N^ (*f*_*i*_ */ X*_*i*_) where *f*_*i*_ is the proportion of the *i*th mixture constituent with dosing point *Xi*. N is the total number of mixture constituents; and if constituents are present in equal numbers, then *fi* = 1/N for all *i* (22). In practice, sequences with alanine at the first position were extracted, their numbers counted, the inverse of their affinities summed, and the values used to determine the harmonic mean. The process was repeated for the remaining 19 amino acids at position 1 and the 20 amino acids for positions 2–9. The results were compiled in a table of 20 x 9 mixture affinities. This table is a model of the results of a screen of a physical positional scanning combinatorial library. The mixtures at each of the 9 positions were then ranked from 1–20 based on their affinity, with 1 corresponding to the amino acid that yielded a mixture with the lowest value and therefore the greatest affinity for the MHCII protein. To compare the peptide repertoire of individual protein genotypes, the ranking values for each of the 9 positions were compared across genotypes using a series of scatterplots (GraphPad Prism 6.1 software). Correlations were performed for each position and **Coefficients of determination (r**^**2**^**) derived from** Pearson correlation coefficient (r) values were recorded. Statistical significance as defined by Graphpad; P values 0.1234 (ns), 0.0332(*), 0.0021(**), 0.0002 (***) and <0.00001(****). To highlight amino acids that ranked highly (i.e., below 5) and were either common to both proteins or found in one protein as opposed to the other, a line for x = y and lines demarking x = 5 and y = 5 were added to the scatter plots.

#### Combination Choice of active amino acids from DQ alleles (step 8 in Fig 1)

Combinations of the amino acids found to be most active at each position of a combinatorial library are used to identify the peptide sequences most likely to be driving activity in the mixtures. We used the amino acids ranked 5 or less and common to 3 IRL DQ proteins to generate (5 x 1 x 3 x 2 x 1 x 2 x 2 x 2 x 2 = 480) peptide sequences. We also wanted to examine the peptide landscape particular to proteins derived from individual alleles, in which case we concentrated on extracting sequences unique to the individual alleles. The amino rankings for the proteins derived from the three confirmed haplotypes DQA1*02DQB1*04 (DQ 2–4), DQA1*01DQB1*08 (DQ1-8) and DQA1*01DQB1*10 (DQ 1–10) were plotted together for each of the nine positions. Amino acids ranked below 5 for each genotype were each given a Hierarchical Value (HV = 1 to 3) determined by their respective occurrence on this plot, first, second or third. For example, F at position 2 was attributed a HV = 1 for DQ 2–4, a HV = 2 for DQ 1–8 and HV = 3 for DQ 1–10; M at position 2 was attributed a HV = 1 for both DQ1-8 and DQ1-10 and had no value at DQ 2–4 as it ranked above 5. Amino acids were chosen for combination based on three criteria,1) they ranked 5 or below, 2) they were attributed HV = 1 and 3) they did not represent a conservative replacement for another amino acid chosen (e.g. Y and F, or S and T, or I and L).

#### Determination of peptide relevance and potential pathogen source (step 9 in Fig 1)

The peptide sequences representing the combinations of amino acids chosen in step 8 for each of the DQ proteins and for those common to all proteins were used in searches of protein databases (Protein Information Resource PIR [39]). Tabulated data of sequence, protein and organism source obtained from these searches were further mined for sequences that were identified in proteins from known marine mammal pathogens.

#### Model PS library analysis for human MHC II: HLA -DP alleles

To determine the utility of the MPSLA, we needed to compare the peptide affinities of the model to the affinities of a physical library. We chose a human MHC protein, HLA DP, where binding affinities had been obtained for physical positional scanning library (26). The physical library explores a 322,687,697,779 (i.e., 19aa^9^) nonomer peptide landscape. A model PS Library was constructed using the human HLA–DP2 alleles [37,52], DPA1*0103:*01*:*01* (https://www.ebi.ac.uk/cgi-bin/ipd/imgt/hla/get_allele.cgi?DPA1*01:*03*:*01*:*01*, Accession number NP_291032) and DPB1*0201 (https://www.ebi.ac.uk/cgibin/ipd/imgt/hla/get_allele.cgi DPB1*02:*01*:*02*:*01*, Accession number CAA26871.1). Sequences of the alleles were obtained from a search of the Immuno Polymorphism Database IPD-IMGT/HLA [53,54]. In this study, human alleles were given the prefix *Hs* to distinguish them from cetacean alleles. Since the model library would be compared to a physical library, cysteines were eliminated. Physical combinatorial libraries omit cysteine from mixtures as this amino acid has the propensity to oxidize and dimerize peptides. A second 7,647 aa list was generated lacking cysteines. The amino acid sequences encoded by *HsDPA* and *HsDPB* were manually supplied to the server. Although the protein sequences derived from these alleles are offered on the NetMHCIIpan 3.1 server, they were entered manually to ensure use of the precise sequence and for consistency as cetacean sequences were supplied manually.

## Supporting information

S1 Fig Sequence of 7,647 amino acids of near equivalent representation of the 20 amino acids. (PDF)

S2 Fig PBR sequences aligned with full length DQA and DQB protein sequences for standard dolphin. (PDF)

S1 Table Calculated binding affinities (nM) in MPSL for DQ 2‐4. The calculated binding affinities for mixtures (nM) derived from the sequences affinities generated by NetMHCIIpan 3.1 for each of the 19 amino acids at nine positions of the core binding peptide for DQ2-4 from the IRL.
(PDF)

S2 Table Calculated binding affinities (nM) in MPSL for DQ 1–1. The calculated binding affinities for mixtures (nM) derived from the sequences affinities generated by NetMHCIIpan 3.1 for each of the 19 amino acids at nine positions of the core binding peptide for DQ1-1 from the IRL.
(PDF)

S3 Table Calculated binding affinities (nM) in MPSL for DQ 1–8. The calculated binding affinities for mixtures (nM) derived from the sequences affinities generated by NetMHCIIpan 3.1 for each of the 19 amino acids at nine positions of the core binding peptide for DQ1-8 from the IRL.
(PDF)

S4 Table Calculated binding affinities (nM) in MPSL for DQ 1–10. The calculated binding affinities for mixtures (nM) derived from the sequences affinities generated by NetMHCIIpan 3.1 for each of the 19 amino acids at nine positions of the core binding peptide for DQ1-10 from the IRL.
(PDF)

S5 Table Calculated binding affinities (nM) in MPSL for DQ *Tursiops truncatus* (used as standard). The calculated binding affinities for mixtures (nM) derived from the sequences affinities generated by NetMHCIIpan 3.1 for each of the 19 amino acids at nine positions of the core binding peptide for *Tursiops truncatus* (used as standard).
(PDF)

S6 Table Calculated binding affinities (nM) in MPSL for DQ *Orcinus orca*. The calculated binding affinities for mixtures (nM) derived from the sequences affinities generated by NetMHCIIpan 3.1 for each of the 19 amino acids at nine positions of the core binding peptide for *Orcinus orca*.
(PDF)

S7 Table Calculated binding affinities (nM) in MPSL for DQ. *Neophocaena phocaenoides*.The calculated binding affinities for mixtures (nM) derived from the sequences affinities generated by NetMHCIIpan 3.1 for each of the 19 amino acids at nine positions of the core binding peptide for *Neophocaena phocaenoides*.
(PDF)

S8 Table Calculated binding affinities (nM) in MPSL for DQ *Physeter macrocephalus*. The calculated binding affinities for mixtures (nM) derived from the sequences affinities generated by NetMHCIIpan 3.1 for each of the 19 amino acids at nine positions of the core binding peptide for *Physeter macrocephalus*.
(PDF)

S9 Table Proteins and pathogens identified from MPSLA for DQ 2–4. The 3,456 sequences derived from amino acids for DQ2-4 were searched for protein matches in the UniProtKB database through the Protein Information Resource (PIR). Sequence matches for proteins originating from reported pathogens in marine mammals are summarized here. Columns listed as (#) refer to numbers identified, or (a) list includes undefined species or proteins. Full details are supplied in S12 Table.
(PDF)

S10 Table Proteins and pathogens identified from MPSLA for DQ 1–10. The 2,586 sequences derived from amino acids for DQ 1–10 were searched for protein matches in the UniProtKB database through the Protein Information Resource (PIR). Sequence matches for proteins originating from reported pathogens in marine mammals are summarized here. Columns listed as (#) refer to numbers identified, or (a) list includes undefined species or proteins. Full details are supplied in S12 Table.
(PDF)

S11 Table Proteins and pathogens identified from MPSLA for DQ 1–8. The 3,888 sequences derived from amino acids for DQ 1-8were searched for protein matches in the UniProtKB database through the Protein Information Resource (PIR). Sequence matches for proteins originating from reported pathogens in marine mammals are summarized here. Columns listed as (#) refer to numbers identified, or (a) list includes undefined species or proteins. Full details are supplied in S12 Table.
(PDF)

S12 Table Combined data from all four searches of proteins and pathogens identified in marine mammals. The sequences derived from amino acids common to the 3 Model Positional scanning libraries and for DQ1-8, DQ1-10 and DQ2-4 were searched for protein matches in the UniProtKB database through the Protein Information Resource (PIR). The search sequences identified in proteins of microbes associated with marine mammals were combined in this table.
(XLSX)
