# Supplementary material for: Bridging immunogenetics and immunoproteomics: Model positional scanning library analysis for Major Histocompatibility Complex class II DQ in Tursiops truncatus
Source: PLoS One. 2018 Aug 2;13(8):e0201299. doi: 10.1371/journal.pone.0201299 (PMC6072028; doi:10.1371/journal.pone.0201299)
Supplement: S1 Fig — (PDF) [file pone.0201299.s001.pdf]

Supp Figure 1

ACDEFGHIKLCDEFGHIKLMDEFGHIKLMNEFGHIKLMNPFGHIKLMNPQGHIKLMNPQRHIKLMNPQRSIKLMNQPQRSKLMNQPQRTS
LMNPQRSTVWMNPQRSTVWYNPQRSTVWYAPQRSTVWYACQRSTVWYACDRSTVWYACDESTVWYACDEFTVWYACDEFGVWYA
CDEFGHWYACDEFGHIYACDEFGHIKLKIHGFEDCAMLKIHGFEDCNMLKIHGFEDPNMLKIHGFEQPNMLKIHGFRQPNMLKIHGSRQP
NMLKIHSRQPQNMLKISTRQPQNMLKWVTSRQPNMLYWVTSRQPNMAYWVTSRQPNCAYWVTSRQPDCAYWVTSRQEDCAYWVTS
RFEDCAYWVTSGFEDCAYWVTHGFEDCAYWVIHGFEDCAYWKIHGFEDCAYADFHKMPRTWCEGILNQSVYDFHKMPRTWAEGILNQ
SVYCFHKMQRTWADGILNPSVYCEHKMPQTWADFILNQRVYCEGKMPRTWADFHLNQSSYCEGIILNQSVYCFHKMPRTWADGILNQS
VYCEHKMQRTWADFILNPSVYCEGKMPQTWADFHLNQRVYCEGIMPRSWADFHKLNQSSYCEGIKMPRTWADFHILNQRVYCEGHKM
PQTWADFGILNPSVYCEFHKMQRTWADEGILNQSVYCDFHKMPRTWACEGILNQSVYADFHKMPRTWPRSWADFHKLNQRVYCEGIK
MPQTWADFHILNPSVYCEGHKMQRTWADFGILNQSVYCEFHKMPWACEGILNQSVYGHIKLCEGILHIKLMDFHKMIKLMNEGILNKLMN
PFHKMPLMNPQGILNQMNPQRHKMQRNPQRSILNPSQPQRSKMPQTPQRTSLNQRVRSTVWMPRSWSTVWYADFHKTVWYACEGIL
VWYACDFHKMWYACDEGILNYACDEFHKMQACDEFGILNPCDEFGHKMPQDEFGHILNQREFGHIKMPRTFGHIKLNQSSFEDCAMPR
SWGFEDCLNQRVHGFEDKMPQTIHGFEILNPSKIHGFHKMQRLKIHGGILNQMLKIHFHKMPNMLKIEGILNQNMLKDFHKMQPNMLCEG
ILRQPNMLNQSSSRQPNKMPRTTSRQPILNQRVTSRQHKMPQWVTSRGILNPYWVTSFHKMQAYWVTEGILNCAYWVDFHKMDCAYW
CEGILEDCAYADFHKWTRPMKHFDAYVSQNLIGECAWTRPMKHFDCYVSQNLIGEDAWTRQMKHFECYVSPNLIGFDAWTQPMKHGE
CYVRQNLIHFDAWTRPMKIGECYSSQNLSQNLIGEDAWTRPMKHFECYVSQNLIGFDAWTRQMKHGECYVSPNLIHFDAWTQPMKIGE
CYVRQNLKHFDAWSRPMIGECYSSQNLHFDAWTRPMKGECYVRQNLIFDAWTQPMKHECYVSPNLIGDAWTRQMKHFCYVSQNLIGE
AWTRPMKHFDYVSQNLIGECWTRPMKHFDADAWSRPMIGECYVRQNLHFDAWTQPMKGECYVSPNLIFDAWTRQMKHECYVSQNLI
GDAWTRPFDYVSQNLIGECLIGECLKIHGMKHFDMLKIHNLIGENMLKIPMKHFPNMLKQNLIGQPNMLRQMKHRQPNMSPNLISRQPNT
QPMKSRQPQVRQNLSTRQPWSRPMWVTSRKHFDAYWVTSLIGECAYWVTMKHFDCAYWVNLIGEDCAYWQMKHFEDCAYPNLIGFE
DCAQPMKHGFEDCRQNLIHGFEDTRPMKIHGFESSQNLKIHGFWSRPMACDEFVRQNLCDEFGTQPMKDEFGHSPNLIEFGHIRQMKH
FGHIKQNLIGGHIKLPMKHFHIKLMNLIGEIKLMNMKHFDKLMNQLIGECLMNPQSSQNLMNPQRTRPMKNPQRSRQNLIPQRSTQPMKH
QRSTVPNLIGRSTVWQMKHFSTVWYNLIGETVWYAMKHFDVWYACLIGECWYACDKHFDAYACDEAGCHDIEKFLCHDIEKFLGMDIEK
FLGMHNEKFLGMHNIPFLGMHNIPKQGMHNIPKQLRHNIPKQLRMSIQKPLQMRNSKPLQMRNTQSLRMSNTPVQWMSNTPVQWRYN
TPVQWRYSAPVQWRYSATCQWRYSATCVDRYSATCVDWESATCVDWEYFTCVDWEYFAGVDWEYFAGCHWEYFAGCHDIYFAGCH
DIEKLFKEIDHCGAMGLFKEIDHCNHMGLFKEIDPINHMGLFKEQKPINHMGLFRLQKPINHMGSMRLQKPINHSNRMQLPKQISQTNRM
QLPKWQVPTNSMRLYRWQVPTNSMASYRWQVPTNCTASYRWQVPDVCTASYRWQEWDVCTASYRFYEWDVCTASGAFYEWDVCT
HCGAFYEWDVIDHCGAFYEWKEIDHCGAFYAMDPFRHTKWCNEQGSIVLYDPFRHTKWMAEQGSIVLYNCFRHTKWMAQDGSIVLYN
CPEHTKWMAPDQFIVLYNCQERGKWMAPDRFTHLYNCQESGSILYNCFRHTKWMAPDGSIVLYNCQEHTKWMAQDRFIVLYNCPESG
KWMAPDQFTHLYNCQERGVIMAPDRFSHWKLYNCQESGSIKWMAPDRFTHIVLYNCQERGHTKWMAPDQFGSIVLYNCPEFRHTKWM
AQDEQGSIVLYNCDPFRHTKWMACNEQGSIVLYAMDPFRHTKWRFSHWKLYNCQERGVIKWMAPDQFTHIVLYNCPESGHTKWMAQD
RFGSIVLYNCQEFRHTKWMAPMACNEQGSIVLYGCHEIGKILLHDIFKHLKMMIEKGLIMLNNKFLHMKNMPPLGMINLPNQQMHNKPMQ
QRRNIPLQNRPSSQKPMQPRQSTPLQNRQTRSVRMSPTRVSWWSATDVFWHYKTCVEWGYIALVDWFYHAKCMWEYGAICLDNYFA
HCKDMEQAGCIDLENFPCHDKEMFPGQDIELFNGQHREKFMGPHRITFLGNHQISKSFMEPDRCSAWGLFNEQDRCVHKGMFPEQDTI
IHLGNFPESKHIKHMGQFRLGKIILHNGQMFLHKKIMHPNEMGLIKLINQDNFMHLKKMQCPENGMILLRLQNPQNSMSSKRMQPPRNTTI
SLRNQQPRVHTKSMRPQQWGVITLSNRPYFWHVKTMSQAEYGWIVLTNCDAFYHWKVMDCCEAGYIWLEADDCFAHYKWKTHRFPD
MAYLVISGQENCAMWKTHRFPDCNYLVISGQEDQAMWKTHRFEPCNYLVISGFQDPAMWKTHGREQCNYLVIHTFRDPAMWKISGSE
QCNYLDCNYLVISGQEDPAMWKTHRFEQCNYLVISGFRDQAMWKTHGSEPCNYLVIHTFQDPAMWKIVGREQCNYLKWHSFRDPAMIS
GSEQCNYLHTFRDPAMWKGREQCNYLVIFQDPAMWKTHEPCNYLVISGDQAMWKTHRFCNYLVISGQEAMWKTHRFPDYLVISGQEN
CWKTHRFPDMAKWHSFRDPAMIVGREQCNYLHTFQDPAMWKGSEPCNYLVIFRDQAMWKTHEQCNYLVISGDPPDYLVISGQENCLLI
KGIEHCGMMKLHKFIDHNNLMILGKEIPPMNKMHLFKQQNPLNIMGLRRQQMPKNHMSSPRNQLPINTSQRPQMPKQVSRTQRNQLPW
WSVRTPSMRKYHWFVDTASLAIYGWEVCTMCKAHYFWDVNDLCIAGYEWQEMDKCHAFYPFNELDICGAQGPFMEKDHCRHQGNFL
EIDTIRHPGMFKESKSIQHNGLFWASCRDPEMFVCRDQENFLGTDQEPFMGKHSEPFNGLHIIRFQGMHKIHKQGNHLIIKGLPHMIKKHL
FMNILKILGMENMKKLHMFNDQLLIMGNEPCQSMSNQPNQLRTNRPPQMRKSRPQQNRLSITQQPRMSKTHVPRNSLTIVGWQSMTKV
HWFYNTLVIWGYEAMVKWHYFADCLWIYGAECCDKYHAFCDDAEWKEIDHCGAFYDIYFAGCHDIECDKYHAFCDDAEWLEADDCFAH
YKEADDCAGCHDDCCEACHDIECDAFYDIEKFAEYGWEKFLGYFWHVFLGMHWGVITGMHNIVHTKSHNIPKTISLRIQKPLSKRMQKPL
QMRLQNPLRMSNQCPENMSNTPQDNFMNTPVQNEMGLPVQWRMFLHKQWRYSLGKIIRYSATKHIKHSATCVIIHLGTCVDWHKGMF
VDWEYGLFNEWEYFAFMEPDYFAGCFLGNHLFKEIEKFMGMGLFKDIELFNHMGLCHDKEPINHMAGCIDQKPINYFAHCRLQKPWEYG
ASMRLQVDWFYSNRMQTCVEWSQTNRSATDVWQVPTRMSPTYRWQVPLQNRASYRWQKPMQCTASYNIPLQDVCTAMHNKPEWD
VCLGMINFYEWDKFLHMGAFYEIEKGLHCGAFHDIFKIDHCGGCHEIKEIDHCNEQGAMDPFDPFRHCNEQGEQGSIDPFRHFRHTKEQ
GSIGSIVLFRHTKHTKWMGSIVLIVLYNHTKWMKWMAPIVLYNLYNCQKWMAPMAPDRLYNCQAMDPFCNEQGCNEQGDPFRHDPFR
HEQGSIEQGSIFRHTKFRHTKGSIVLGSIVLHTKWMHTKWMIVLYNIVLYNKWMAPKWMAPLYNCQLYNCQMAPDRMAPDRLYNCQLY
NCQKWMAPKWMAPIVLYNIVLYNHTKWMHTKWMGSIVLGSIVLFRHTKFRHTKEQGSIEQGSIDPFRHDPFRHCNEQGCNEQGAMDPF
LYNCQMAPDRKWMAPLYNCQIVLYNKWMAPHTKWMIVLYNGSIVLHTKWMFRHTKGSIVLEQGSIFRHTKDPFRHEQGSICNEQGDPF
RHAMDPFCNEQGKYHAFGCHEILWIYGHDIFKMVKWHIEKGLNTLVIKFLHMQSMTKLGMINPRNSLMHNKPQQPRMNIPLQRPQQNQK
PMQTNRPPPLQNRSMSNQRMSPTLLIMGSATDVMKKLHTCVEWNILKIVDWFYPHMIKWEYGAQGNHLYFAHCRFQGMAGCIDSEPFN
CHDKETDQEPDIELFVCRDQEKFMGWASCRFLGNHSKSIQFMEPDTIRHPGLFNERHQGNHKGMFQGPFMIIHLGPFNELKHIKHQEMD
KLGKIINDLCIMFLHKMCKAHNEMGLLAIYGQDNFMKYHWFQCPENWWSVRRLQNPVSRTQSKRMQTSQRPTISLRSSPRNVHTKSRR
QQMWGVITQQNPLYFWHVPPMNKAEYGWNNLMICDAFYMMKLHDCCEALLIKGEADDCYLVISWKTHRAMWKTYLVISCNYLVAMWK

TDPAMWCNYLVEQCNYDQAMWFRDQAEPCNYGSEPCFQDPAHTFQDGREQCIVGREHTFRDKWHSFISGSEWKTHRYLVISYLVISA
MWKTAMWKTCNYLVCNYLVDPAMWDQAMWEQCNYEPCNYFRDQAFQDPAGSEPCGREQCHTFQDHTFRDIVGREISGSEKWHSFK
WHSFISGSEIVGREHTFRDHTFQDGREQCGSEPCFQDPAFRDQAEPCNYEQCNYDQAMWDPAMWCNYLVCNYLVAMWKTAMWKTY
LVISYLVISWKTHRISGSEKWHSFHTFRDIVGREGREQCHTFQDFQDPAGSEPCEPCNYFRDQADQAMWEQCNYCNYLVDPAMWAMW
KTCNYLVYLVISAMWKTWKTHRYLVISYFAGCLLIKGWEYFAMMKLHVDWEYNNLMITCVDWPPMNKSATCVQQNPLRYSATRRQQMQ
WRYSSSPRNPVQWRTSQRPNTPVQVSRTQMSNTPWWSVRLRMSNKYHWFKPLQMLAIYGIQKPLMCKAHHNIPKNDLCIGMHNIQEM
DKFLGMHPFNELEKFLGQGPFMDIEKFRHQGNCHDIETIRHPAGCHDSKSIQKEIDHWASCRIDHCGVCRDQHCGAFTDQEPGAFYESE
PFNFYEWDRFQGMEWDVCQGNHLDVCTAPHMIKCTASYNILKIASYRWMKKLHYRWQVLLIMGWQVPTSMSNQSQTNRTNRPPSNR
MQRPQQNSMRLQQQPRMRLQKPPRNSLQKPINQSMTKPINHMNTLVINHMGLMVKWHMGLFKLWIYGLFKEIKYHAFKEIDHKYHAFLF
KEILWIYGMGLFKMVKWHNHMGLNTLVIPINHMQSMTKQKPINPRNSLRLQKPQQPRMRMQLPRPQQNTNRMQTNRPPVPTNSSMSN
QWQVPTLLIMGYRWQVMKKLHASYRWNILKICTASYPHMIKDVCTAQGNHLEWDVCRFQGMFYEWDSEPFNGAFYETDQEPHCGAFV
CRDQIDHCGWASCRCHDIESKSIQDIEKFTIRHPEKFLGRHQGNFLGMHQGPFMGMHNIPFNELHNIPKQEMDKIPKQLNDLCIKPLQMM
CKAHLQMRNLAIYGMSNTPKYHWFNTPVQWWSVRPVQWRVSRTQQWRYSTSQRPRYSATSSPRNSATCVRRQQMTCVDWQQNPL
VDWEYPPMNKWEYFANNLMIYFAGCMMKLHAGCHDLLIKGTHRFPYLVISVISGQAMWKTWKTHRCNYLVYLVISDPAMWAMWKTEQ
CNYCNYLVFRDQADPAMWGSEPCEQCNYHTFQDFRDPAIVGREGSEQCKWHSFVISGQWKTHRTCNYLVCNYLVDQAMWDQAMWE
PCNYEPCNYFQDPAFQDPAGREQCGREQCHTFRDHSFRDISGSEGSEQCKWHSFFRDPAIVGREEQCNYHTFQDDPAMWGSEPCCN
YLVFRDQAAMWKTEQCNYYLVISDPAMWWKTHRCNYLVVISGQAMWKTTHRFPYLVISHSFRDISGSEGREQCHTFRDFQDPAGREQ
CEPCNYFQDPADQAMWEPCNYCNYLVDQKTWKTHRYLVISVISGQWKTHRIKGIEEADDCKLHKFDCCEALMILGCDAFYMNKMHAEY
GWNPLNIYFWHVQQMPKWGVITPRNQLVHTKSQRPQMTISLRRTQRNSKRMQSVRTPRLQNPHWFVDQCPENIYGWEQDNFMKAHY
FNEMGLLCIAGMFLHKMDKCHLGKIINELDIKHIKHPFMEKIIHLGQGNFLHKGMFRHPGMGLFNESIQHNFMEPDSCRDPFLGNHRDQE
NEKFMGQEPFMDIELFPFNGLCHDKEQGMHKAGCIDNHLIIYFAHCMIKKHWEYGALKILGVDWFYKLHMFTCVEWIMGNESATDVSNQ
PNRMSPTRPPQMPLQNRQQNRLQKPMQPRMSKNIPLQNSLTIMHNKPMTKVHLGMINLVIWGKFLHMKWHYFIEKGLIYGAEHDIFKHA
FCDGCHEIAPMAPDRLYNCQNCQESMAPDREQGSIAMDPFFRHTKCNEQGGSIVLDPFRHHTKWMEQGSIIVLYNFRHTKKWMAQGSI
VLLYNCPHTKWMMAPDQIVLYNNCQERKWMAPPDRFSCQNCQESRHDPFRHCNEQGRFSLYNCQNCQERKWMAPMAPDQIVLYNLY
NCPHTKWMKWMAQGSIVLIVLYNFRHTKHTKWMEQGSIGSIVLDPFRHFRHTKCNEQGEQGSIAMDPFHEIGKKEIDHIFKHLIDHCGKG
LIMHCGAFLHMKNGAFYEMINLPFYEWDNKPMQEWDVCPLQNRDVCTAPMQPRCTASYQNRQTASYRWSPTRVYRWQVTDVFWWQ
VPTVEWGYSQTNRWFYHASNRMQYGAICSMRLQAHCKDRLQKPCIDLEQKPINDKEMFPINHMELFNGNHMGLFMGPHMGLFKGNHQ
ILFKEIEPDRCYFAGCFNEQDWEYFAGMFPEVDWEYHLGNFTCVDWIKHMGSATCVKIILHRYSATLHKKIQWRYSMGLIKPVQWRNFM
HLNTPVQPENGMMSNTPQNPQNLRMSNRMQPPKPLQMSLRNQIQKPLTKSMRHNIPKVITLSGMHNIWHVKTFLGMHYGWIVEKFLGA
FYHWDIEKFCEAGYCHD IEDDCFAAGCHD
