# Supplementary material for: Bridging immunogenetics and immunoproteomics: Model positional scanning library analysis for Major Histocompatibility Complex class II DQ in Tursiops truncatus
Source: PLoS One. 2018 Aug 2;13(8):e0201299. doi: 10.1371/journal.pone.0201299 (PMC6072028; doi:10.1371/journal.pone.0201299)
Supplement: S2 Fig — (PDF) [file pone.0201299.s002.pdf]

DQA

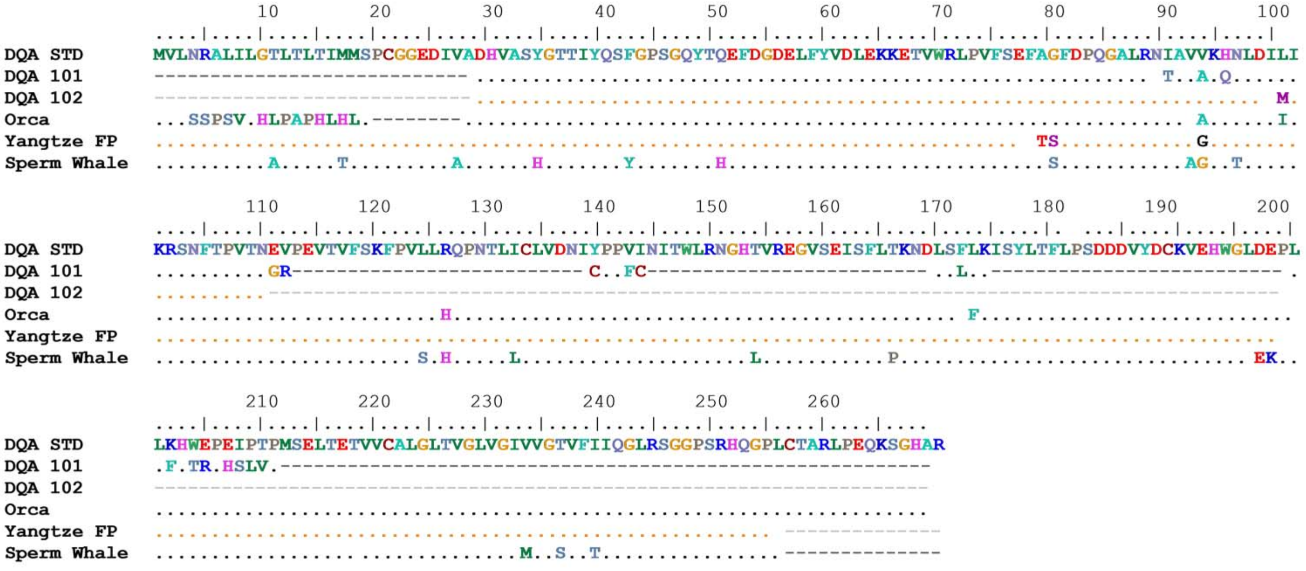
DQA STD
DQA 101
DQA 102
Orca
Yangtze FP
Sperm Whale
MVLNRALILGTLTLTIMMSPCGGEDIVADHVASYGTTIYQSFGPSGQYTQEFDGDELFYVDLEKKETVWRLPVFSEFAGFDPQGALRNIAVVKHNLDILI
KRSNFTPVTNEVPEVTVFSKFPVLLRQPNTLICLVDNIYPPVINITWLRNGHTVREGVSEISFLTKNDLSFLKISYLTFLPSDDDVYDCKVEHWGLDEPL
LKHWEPEIPTPMSELTETVVCALGLTVGLVGIVVGTVFIIQGLRSGGPSRHQGPLCTARLPEQKSGHAR

DQB

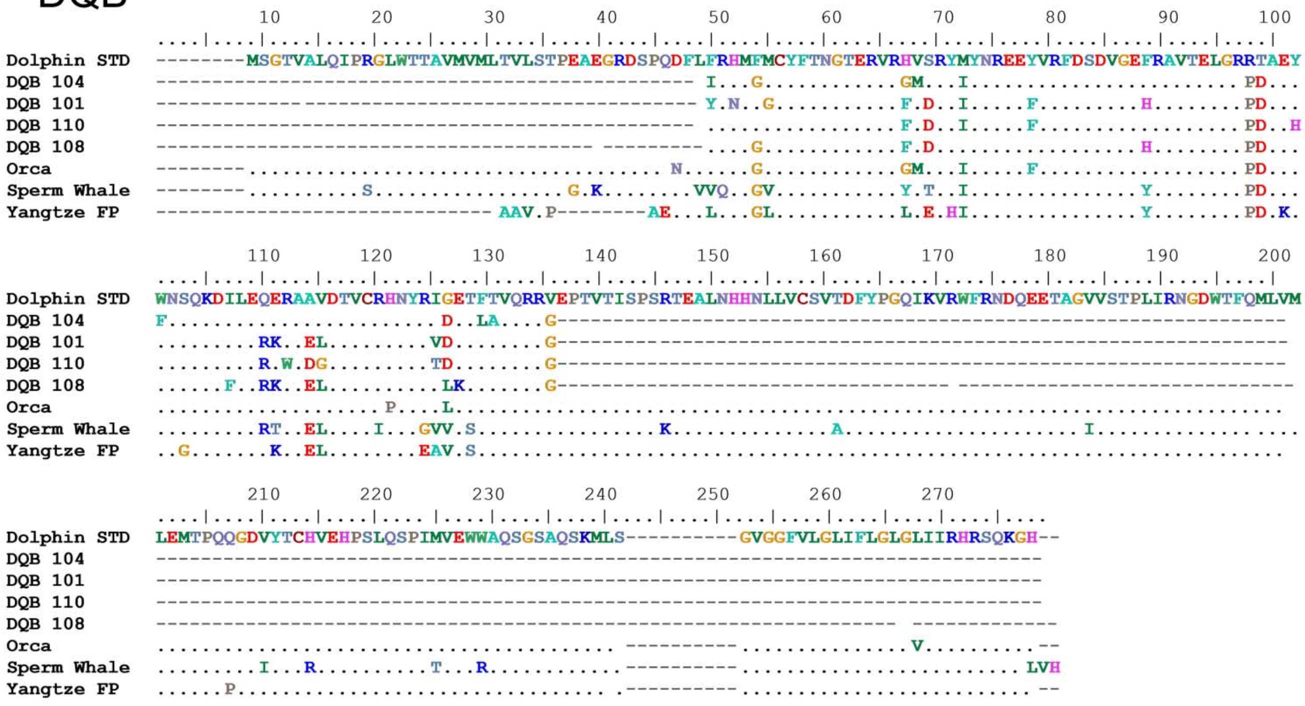
Dolphin STD
DQB 104
DQB 101
DQB 110
DQB 108
Orca
Sperm Whale
Yangtze FP
--------MSGTVALQIPRGLWTTAVMVMLTVLSTPEAEGRDSPQDFLFRHMFMCYFTNGTERVRHVSRYMYNREEYVRFDSDVGEFRAVTELGRRTAEY
WNSQKDILEQERAAVDTVCRHNYRIGETFTVQRRVEPTVTISPSRTEALNHHNLLVCSVTDFYPGQIKVRWFRNDQEETAGVVSTPLIRNGDWTFQMLVM
LEMTPQQGDVYTCHVEHPSLQSPIMVEWWAQSGSAQSKMLS----------GVGGFVLGLIFLGLGLIIRHRSQKGH--
