# Supplementary material for: Bridging immunogenetics and immunoproteomics: Model positional scanning library analysis for Major Histocompatibility Complex class II DQ in Tursiops truncatus
Source: PLoS One. 2018 Aug 2;13(8):e0201299. doi: 10.1371/journal.pone.0201299 (PMC6072028; doi:10.1371/journal.pone.0201299)
Supplement: S1 Table — The calculated binding affinities for mixtures (nM) derived from the sequences affinities generated by NetMHCIIpan 3.1 for each of the 19 amino acids at nine positions of the core binding peptide for DQ2-4 from the IRL. (PDF) [file pone.0201299.s003.pdf]

Supp Table 1: Calculated binding affinities (nM) in MPSL for DQ 2-4

Column1	Pos1	Pos2	Pos3	Pos4	Pos5	Pos6	Pos7	Pos8	Pos9
A	3621.658	3473.315	2441.761	3094.573	2867.609	2202.149	3152.641	2674.464	2156.086
C	4306.358	5467.358	3780.311	5545.272	3703.158	5600.483	4176.665	4525.453	4006.265
D	4602.888	3608.538	4419.478	2433.007	2963.093	2404.46	2573.377	3670.588	2719.843
E	5533.611	3998.17	4600.476	3980.952	3573.67	4663.58	3628.926	2933.755	4045.864
F	3450.473	2191.473	3008.045	2673.185	2643.441	3192.556	3116.158	3459.335	3585.304
G	5159.475	3952.402	3394.184	3379.551	4042.136	3816.571	5250.011	4729.55	4956.068
H	4182.269	3301.477	5426.83	4749.791	4603.904	5644.765	4956.835	4777.372	4943.315
I	3534.107	2913.593	3648.035	3213.888	3452.54	3069.996	3500.83	2841.121	3680.629
K	5131.011	5089.501	5326.449	6158.445	5205.284	7398.508	4820.889	4529.749	4297.907
L	3544.233	2526.476	3577.25	3158.3	3351.104	3352.297	3007.858	2741.112	3124.753
M	4377.221	2885.916	3766.571	4011.407	3980.403	5062.009	2821.151	3496.163	2913.414
N	6408.525	4368.949	4368.094	3882.728	3911.959	3758.754	4094.817	3628.594	4227.856
P	7300.143	6916.529	6731.387	5868.092	3320.643	3913.641	4312.228	3837.296	4161.865
Q	5909.318	5457.796	4253.132	4155.395	3656.202	3662.957	3414.952	3566.222	4646.001
R	5980.709	5190.019	4359.348	6947.541	4917.326	4973.282	6276.308	4707.545	5697.469
S	5689.218	3931.013	4040.685	4285.892	3289.429	3504.483	3474.904	4541.961	5666.932
T	4107.827	4313.347	3959.604	4396.586	3943.106	3771.847	3484.984	4357.719	5234.289
V	3033.34	3385.232	1889.781	2731.215	2210.159	2945.376	3068.162	2791.193	2932.244
W	2476.558	2512.876	2368.557	3502.955	2887.121	3645.569	3514.987	2133.861	3186.894
Y	1981.173	2513.112	2462.875	2596.196	2790.888	3667.058	3201.245	3211.174	2997.058
