# Supplementary material for: Bridging immunogenetics and immunoproteomics: Model positional scanning library analysis for Major Histocompatibility Complex class II DQ in Tursiops truncatus
Source: PLoS One. 2018 Aug 2;13(8):e0201299. doi: 10.1371/journal.pone.0201299 (PMC6072028; doi:10.1371/journal.pone.0201299)
Supplement: S2 Table — The calculated binding affinities for mixtures (nM) derived from the sequences affinities generated by NetMHCIIpan 3.1 for each of the 19 amino acids at nine positions of the core binding peptide for DQ1-1 from the IRL. (PDF) [file pone.0201299.s004.pdf]

**Supp Table 2: Calculated binding affinities (nM) in MPSL for DQ 1-1**

| Column1 | Pos1 | Pos2 | Pos3 | Pos4 | Pos5 | Pos6 | Pos7 | Pos8 | Pos9 |
|---|---|---|---|---|---|---|---|---|---|
| A | 5842.462 | 4473.23 | 2725.744 | 3785.842 | 2744.332 | 2595.663 | 3760.225 | 3841.449 | 2555.472 |
| C | 5775.951 | 5936.711 | 5188.135 | 6626.406 | 4525.505 | 8001.906 | 5502.159 | 5857.402 | 5265.993 |
| D | 5867.878 | 5468.635 | 4388.257 | 3414.704 | 4608.556 | 4438.793 | 3921.714 | 5414.178 | 4964.909 |
| E | 5409.935 | 5538.234 | 5698.273 | 5731.955 | 4851.69 | 6852.81 | 5796.485 | 5817.687 | 5624.723 |
| F | 3521.296 | 3267.093 | 5450.572 | 5027.34 | 5884.003 | 7394.841 | 5503.366 | 6524.114 | 6373.493 |
| G | 6012.296 | 3721.322 | 3651.222 | 3440.688 | 3918.941 | 3493.834 | 4630.164 | 5615.118 | 4438.904 |
| H | 5611.993 | 4146.439 | 4488.936 | 6660.853 | 5546.562 | 7498.155 | 6267.027 | 5028.213 | 5511.043 |
| I | 4141.813 | 3240.031 | 4181.033 | 3720.824 | 3601.676 | 4607.942 | 3324.086 | 2935.359 | 3812.568 |
| K | 5161.22 | 5796.901 | 5270.375 | 8072.786 | 5148.035 | 8363.066 | 4800.969 | 5190.484 | 3701.537 |
| L | 4472.256 | 3731.287 | 4845.253 | 4440.413 | 4107.768 | 5008.123 | 3578.637 | 3901.869 | 3407.086 |
| M | 5168.873 | 2733.379 | 4057.601 | 4222.86 | 4687.065 | 4814.499 | 3009.454 | 3542.436 | 3896.808 |
| N | 6035.998 | 4966.707 | 5123.356 | 4356.233 | 4274.28 | 3751.017 | 4048.233 | 4005.895 | 4290.235 |
| P | 7565.365 | 5946.706 | 5698.343 | 4044.913 | 2629.63 | 3498.024 | 3314.14 | 4121.461 | 5554.618 |
| Q | 6493.663 | 4515.156 | 5471.416 | 3733.942 | 3904.514 | 4553.126 | 4202.796 | 3720.527 | 4822.051 |
| R | 6548.037 | 3681.674 | 4671.618 | 5393.617 | 4913.102 | 5636.584 | 4549.932 | 3870.715 | 4791.875 |
| S | 5099.808 | 2899.969 | 3011.525 | 2748.61 | 2478.417 | 2386.807 | 2362.279 | 3495.065 | 3388.806 |
| T | 5256.241 | 4325.088 | 5065.322 | 3460.997 | 2432.455 | 3191.557 | 3254.451 | 4286.512 | 4632.74 |
| V | 3682.349 | 3929.185 | 2463.838 | 3047.776 | 3589.173 | 4324.791 | 3499.414 | 2527.378 | 2746.238 |
| W | 2420.921 | 3961.238 | 3983.132 | 6403.691 | 5590.1 | 6148.284 | 5340.661 | 2843.488 | 2880.664 |
| Y | 2714.383 | 3104.156 | 3594.492 | 5132.656 | 5467.487 | 6496.759 | 5655.094 | 4798.333 | 3854.068 |
