# Supplementary material for: Bridging immunogenetics and immunoproteomics: Model positional scanning library analysis for Major Histocompatibility Complex class II DQ in Tursiops truncatus
Source: PLoS One. 2018 Aug 2;13(8):e0201299. doi: 10.1371/journal.pone.0201299 (PMC6072028; doi:10.1371/journal.pone.0201299)
Supplement: S3 Table — The calculated binding affinities for mixtures (nM) derived from the sequences affinities generated by NetMHCIIpan 3.1 for each of the 19 amino acids at nine positions of the core binding peptide for DQ1-8 from the IRL. (PDF) [file pone.0201299.s005.pdf]

Supp Table 3: Calculated binding affinities (nM) in MPSL for DQ 1-8

Column1	Pos1	Pos2	Pos3	Pos4	Pos5	Pos6	Pos7	Pos8	Pos9
A	2894.9	2089.459	1438.348	1944.429	1511.42	1576.335	1756.85	1704.296	1376.391
C	2830.327	2620.236	2719.792	3201.379	2199.5	4091.521	2868.673	2824.166	2815.889
D	3084.427	2927.642	2760.198	2086.846	2491.68	2482.046	2150.758	2780.638	2417.291
E	2985.931	2941.337	3062.038	3007.646	3000.788	3826.526	3022.603	2496.679	2675.045
F	1903.794	1819.639	2227.472	2190.43	2301.782	2973.264	2281.599	2952.315	2693.699
G	3271.216	1851.125	1840.86	1754.407	1995.185	2010.827	1842.004	2319.221	2318.959
H	2467.511	1947.271	2287.26	2832.752	2453.913	3231.556	2611.153	2339.05	2152.601
I	1933.184	1535.998	1681.017	1654.476	1414.216	1707.18	1640.899	1694.551	1931.265
K	2613.402	2506.336	2284.314	3027.892	2203.438	3214.236	2158.448	2248.871	1558.9
L	1840.077	1581.427	2054.702	1577.016	1819.111	1798.527	1533.999	1854.671	1698.027
M	2286.36	1488.785	2030.05	1915.434	2042.83	1978.558	1564.975	1827.103	1750.202
N	2862.646	2333.008	2364.522	2212.115	2418.763	2069.194	2188.539	1885.696	2118.784
P	3904.442	2329.673	3325.118	2200.607	1830.258	1929.618	1994.464	2114.652	2430.06
Q	3224.107	2503.876	2914.499	2160.584	2310.519	2050.964	2133.464	1865.28	2175.147
R	3140.529	1986.39	2463.673	2907.617	2314.791	2427.874	2314.148	1951.356	2413.675
S	2936.655	1578.863	1528.49	1502.337	1481.485	1371.125	1438.338	1754.845	1813.967
T	2202.28	1961.924	2424.753	1806.134	1560.127	1625.071	1788.829	2139.174	2007.643
V	1640.296	1675.998	1244.793	1209.075	1282.902	1610.065	1651.289	1408.63	1675.399
W	1392.953	1570.844	1803.159	2132.914	2190.836	2151.511	2147.45	1594.775	1543.302
Y	1338.433	1607.486	1503.501	1874.59	2087.485	2537.318	2184.018	1739.449	1821.526
