# Supplementary material for: Bridging immunogenetics and immunoproteomics: Model positional scanning library analysis for Major Histocompatibility Complex class II DQ in Tursiops truncatus
Source: PLoS One. 2018 Aug 2;13(8):e0201299. doi: 10.1371/journal.pone.0201299 (PMC6072028; doi:10.1371/journal.pone.0201299)
Supplement: S4 Table — The calculated binding affinities for mixtures (nM) derived from the sequences affinities generated by NetMHCIIpan 3.1 for each of the 19 amino acids at nine positions of the core binding peptide for DQ1-10 from the IRL. (PDF) [file pone.0201299.s006.pdf]

Supp Table 4: Calculated binding affinities (nM) in MPSL for DQ 1-10

Column1	Pos1	Pos2	Pos3	Pos4	Pos5	Pos6	Pos7	Pos8	Pos9
A	5949.908	5601.154	3730.146	4778.529	4131.149	3336.287	4283.016	4659.166	3369.866
C	6624.492	7159.248	6285.582	7787.259	5503.505	9465.937	6855.803	6742.118	6874.88
D	6654.068	5841.409	6449.34	4228.81	5674.016	5064.162	5049.732	6589.995	5906.407
E	7112.024	6440.688	6918.728	6504.163	6052.486	8289.221	6641.624	6571.799	7244.515
F	5096.765	4115.22	5848.905	6166.885	6577.266	7586.459	6491.355	6839.704	7191.344
G	6678.198	5194.712	4385.933	4292.425	4612.432	4574.278	6220.474	7086.861	6131.594
H	6874.039	5335.791	6894.882	7577.484	7071.051	8721.162	7653.663	6876.276	7109.725
I	5132.702	3784.235	4792.541	4535.096	4508.629	5170.997	4154.221	3747.106	4931.709
K	7109.05	7223.81	6950.592	9790.304	7367.367	10341.89	6361.126	6914.418	4908.426
L	5284.472	4508.682	5881.535	5066.619	4762.492	5902.923	4289.482	4390.766	4484.535
M	6258.425	3715.416	5361.957	5595.944	5953.651	6542.376	4153.83	4776.733	4837.41
N	7182.955	6289.818	5990.697	5478.816	5669.254	4617.322	5499.898	5404.845	6128.549
P	9966.273	8604.748	7424.896	6287.552	3968.86	5029.839	5784.676	5828.571	7168.217
Q	8487.713	6349.187	6710.026	5342.791	4938.448	6241.202	5118.819	5490.16	6846.781
R	8115.959	6095.503	6130.388	7846.769	6956.81	7301.391	7454.49	5938.373	7107.818
S	6142.306	4358.965	4178.585	4131.994	3886.336	3665.441	3697.63	4927.307	5566.116
T	6287.183	5391.738	6561.359	5488.651	4311.179	4944.703	4350.469	5830.489	6480.833
V	4400.145	4852.198	3256.916	4356.935	4382.624	4867.724	4559.885	3464.914	3504
W	3526.042	5055.421	5133.519	7432.311	6356.264	7419.817	6507.133	3438.317	3916.335
Y	3748.743	4282.859	4747.43	5545.601	5847.014	7120.385	5756.928	5634.132	4960.247
