# Supplementary material for: Bridging immunogenetics and immunoproteomics: Model positional scanning library analysis for Major Histocompatibility Complex class II DQ in Tursiops truncatus
Source: PLoS One. 2018 Aug 2;13(8):e0201299. doi: 10.1371/journal.pone.0201299 (PMC6072028; doi:10.1371/journal.pone.0201299)
Supplement: S5 Table — The calculated binding affinities for mixtures (nM) derived from the sequences affinities generated by NetMHCIIpan 3.1 for each of the 19 amino acids at nine positions of the core binding peptide for Tursiops truncatus (used as standard). (PDF) [file pone.0201299.s007.pdf]

Supp Table 5: Calculated binding affinities (nM) in MPSL for DQ *Tursiops truncatus* (used as standard)

Column1	Pos1	Pos2	Pos3	Pos4	Pos5	Pos6	Pos7	Pos8	Pos9
A	1131.061	1028.493	678.415	843.7557	719.9984	626.5251	844.1687	934.0717	672.0238
C	1447.618	2103.872	1373.213	1834.396	1121.1	2272.943	1286.498	1558.803	1380.46
D	1782.512	1104.384	1207.145	755.9937	836.3921	810.1278	795.0864	1148.098	866.1353
E	1995.354	1246.983	1307.744	1402.093	1030.282	1497.67	1097.561	951.8582	1299.99
F	978.5507	634.5671	989.2312	859.5818	950.5554	1092.922	917.5946	1169.865	1121.374
G	1566.176	1133.625	1011.481	922.4293	1177.054	1107.888	1644.535	1654.786	1684.652
H	1665.991	1097.653	2078.872	1644.499	1661.825	1908.515	1895.282	1908.446	1969.782
I	1250.258	1023.936	1313.34	1095.809	1219.547	1213.799	1101.682	882.3084	1244.522
K	2104.918	2074.314	2255.179	2550.35	2066.367	3191.038	1743.334	1743.603	1332.588
L	1297.432	935.1706	1383.213	1289.503	1361.556	1323.421	1055.854	898.2261	1050.611
M	1765.326	824.9412	1475.616	1192.67	1294.657	1909.396	882.6437	1079.815	1075.025
N	2476.915	1627.736	1871.234	1515.819	1608.508	1349.357	1393.643	1269.661	1459.498
P	3784.436	2458.071	2381.712	1743.37	951.6864	1075.408	1477.171	1311.435	1638.639
Q	2857.2	1905.076	1491.149	1355.118	1146.376	1389.084	1265.108	1160.896	1931.437
R	2360.764	1905.014	1446.569	3081.186	1937.701	1622.186	2264.319	1566.492	2069.54
S	1644.096	1229.489	1153.033	1147.651	933.2757	1042.997	1135.938	1550.575	1973.482
T	1532.363	1394.301	1534.234	1451.22	1050.273	1419.493	1096.71	1418.745	1897.63
V	1072.988	1095.013	632.0066	1042.017	806.844	1115.514	920.7072	846.4546	837.4189
W	694.0888	764.4326	800.3758	1392.704	1010.336	1268.122	1144.399	661.5213	835.2265
Y	660.5709	729.4015	764.6401	933.7644	970.5411	1271.626	1056.338	1055.526	953.3972
