# Supplementary material for: Bridging immunogenetics and immunoproteomics: Model positional scanning library analysis for Major Histocompatibility Complex class II DQ in Tursiops truncatus
Source: PLoS One. 2018 Aug 2;13(8):e0201299. doi: 10.1371/journal.pone.0201299 (PMC6072028; doi:10.1371/journal.pone.0201299)
Supplement: S6 Table — The calculated binding affinities for mixtures (nM) derived from the sequences affinities generated by NetMHCIIpan 3.1 for each of the 19 amino acids at nine positions of the core binding peptide for Orcinus orca. (PDF) [file pone.0201299.s008.pdf]

Supp Table 6: Calculated binding affinities (nM) in MPSL for DQ *Orcinus orca*

Column1	Pos1	Pos2	Pos3	Pos4	Pos5	Pos6	Pos7	Pos8	Pos9
A	1809.786	1882.09	1365.521	1742.131	1423.369	1232.951	1568.284	1265.912	1299.351
C	2258.557	3228.54	2300.932	3276.456	2123.364	3285.334	2130.117	2774.487	2619.48
D	2810.54	2007.448	2486.513	1413.841	1618.79	1205.535	1421.888	1776.344	1502.623
E	3971.353	2348.625	2916.966	2370.016	1912.069	2362.912	1979.707	1573.755	2454.006
F	1971.504	1289.427	1373.16	1312.4	1580.522	1710.634	1637.722	1811.768	1717.129
G	2747.379	2145.257	1800.288	1772.572	2308.48	1985.182	3116.85	2672.091	2996.834
H	2442.027	1779.452	3125.102	2598.934	2195.24	3084.953	2640.657	2689.453	2701.277
I	1686.178	1353.124	1712.617	1683.329	1828.997	1684.712	2088.45	1648.887	1969.543
K	3092.657	2674.195	3122.613	3429.886	2913.977	3759.808	2780.875	2366.535	2305.431
L	1665.606	1298.945	1707.715	1549.657	1922.066	1720.858	1711.071	1450.14	1679.542
M	2372.994	1617.947	2060.153	2239.537	2039.048	2670.397	1604.328	1788.784	1346.945
N	3846.515	2274.247	2622.459	2199.174	2303.905	2024.957	2429.893	2206.282	2403.172
P	4326.036	5197.82	3961.347	3658.14	2071.987	2262.953	2182.969	2121.232	2366.35
Q	4352.339	3181.619	2578.138	2476.485	1934.995	2324.663	1745.061	1992.508	2804.256
R	3959.417	3216.098	2656.051	3640.646	2631.859	2947.488	3605.079	2513.481	3334.067
S	2998.868	2066.252	2256.77	2522.559	1880.796	1974.996	2001.243	2800.414	3633.546
T	2520.552	2424.663	2150.009	2624.898	2426.68	2053.964	2033.486	2213.701	3060.637
V	1453.583	1423.504	1016.733	1407.838	1146.437	1432.511	1739.044	1592.608	1509.466
W	1442.951	1490.335	1254.837	1583.79	1291.832	1698.176	1703.963	1128.466	1601.485
Y	1069.154	1308.419	1215.926	1273.981	1277.209	1896.732	1626.739	1744.441	1624.973
