# Supplementary material for: Bridging immunogenetics and immunoproteomics: Model positional scanning library analysis for Major Histocompatibility Complex class II DQ in Tursiops truncatus
Source: PLoS One. 2018 Aug 2;13(8):e0201299. doi: 10.1371/journal.pone.0201299 (PMC6072028; doi:10.1371/journal.pone.0201299)
Supplement: S7 Table — Neophocaena phocaenoides.The calculated binding affinities for mixtures (nM) derived from the sequences affinities generated by NetMHCIIpan 3.1 for each of the 19 amino acids at nine positions of the core binding peptide for Neophocaena phocaenoides. (PDF) [file pone.0201299.s009.pdf]

Supp Table 7: Calculated binding affinities (nM) in MPSL for DQ *Neophocaena phocaenoides*

Column1	Pos1	Pos2	Pos3	Pos4	Pos5	Pos6	Pos7	Pos8	Pos9
A	927.8525	766.9142	537.4887	706.5051	439.0394	512.9451	518.8536	500.584	530.3664
C	940.6855	951.8627	990.3961	1254.929	777.1276	1513.093	1117.87	1005.015	1370.74
D	1065.673	935.7596	1323.689	699.1512	701.4269	807.742	716.4248	1082.235	834.375
E	1208.23	1003.455	1493.03	934.8286	1111.521	1519.361	1004.641	962.0808	1062.079
F	712.1897	653.6381	501.2475	708.0078	839.9533	894.3151	592.3894	803.1408	750.8771
G	1360.011	665.8372	560.5838	592.7609	624.5711	591.2742	806.5915	915.8832	928.1497
H	752.8596	597.9275	679.4167	775.8114	610.3952	1025.89	683.1279	658.8327	533.423
I	429.5332	335.4991	398.1927	449.3989	482.6957	520.5463	545.0217	527.3007	506.2175
K	910.7201	623.8836	612.1952	908.6821	674.5774	755.7168	629.0861	568.7866	430.0323
L	414.3455	408.5696	460.8306	411.3818	487.1678	494.8776	429.435	476.4447	439.3355
M	590.8654	571.6216	521.5206	478.6931	688.7746	512.3982	444.5653	502.909	392.0446
N	1096.729	681.4034	649.4024	639.4642	575.2374	517.7186	776.6732	651.7082	766.0999
P	1232.002	1091.099	1094.269	919.9004	611.5737	561.0192	693.5502	745.7815	864.3657
Q	1176.197	786.2841	777.7018	613.547	615.9477	624.3476	556.2376	566.4928	785.0512
R	1337.663	592.6207	695.6935	870.7756	698.7882	726.0022	660.3675	457.6693	683.2775
S	1011.681	533.1418	487.3978	480.9681	463.4491	449.0572	513.4564	655.4754	692.6561
T	800.7179	631.9815	700.0649	644.6907	664.654	407.2992	623.1111	693.9184	702.5435
V	466.8926	381.9504	388.7896	407.6047	424.16	538.4922	597.0757	471.7449	550.9233
W	572.9013	673.9976	607.6605	585.0058	660.0692	725.4737	650.5403	489.4848	429.482
Y	496.1744	533.262	490.6804	564.006	582.0191	837.4741	621.262	657.6894	582.9244
