# Supplementary material for: Bridging immunogenetics and immunoproteomics: Model positional scanning library analysis for Major Histocompatibility Complex class II DQ in Tursiops truncatus
Source: PLoS One. 2018 Aug 2;13(8):e0201299. doi: 10.1371/journal.pone.0201299 (PMC6072028; doi:10.1371/journal.pone.0201299)
Supplement: S8 Table — The calculated binding affinities for mixtures (nM) derived from the sequences affinities generated by NetMHCIIpan 3.1 for each of the 19 amino acids at nine positions of the core binding peptide for Physeter macrocephalus. (PDF) [file pone.0201299.s010.pdf]

Supp Table 8: calculated binding affinities (nM) in MPSL for DQ *Physeter macrocephalus*

Column1	Pos1	Pos2	Pos3	Pos4	Pos5	Pos6	Pos7	Pos8	Pos9
A	3042.22	2649.185	1973.34	2395.988	1689.617	1889.77	1979.285	2102.338	1917.234
C	3484.338	3349.131	3651.577	4280.171	2958.265	5213.482	3443.874	3493.163	3863.103
D	3176.827	3533.889	3729.758	2455.318	3040.049	2822.287	2614.776	3625.83	3403.297
E	3297.003	3318.021	3667.176	3475.003	3346.169	4868.147	3776.949	3523.85	3888.247
F	3062.473	2709.633	2770.2	3170.402	3836.637	4448.679	3381.603	3536.821	3676.994
G	3375.111	2493.454	2005.564	2231.362	2083.462	2441.134	2650.259	3156.976	3001.941
H	3209.289	2428.288	3115.193	3673.367	3390.014	4453.858	3322.283	3140.382	2902.787
I	1982.651	1747.176	1905.047	2048.497	1908.692	2095.379	2023.641	2119.33	2207.365
K	3345.276	2949.137	2806.94	3519.882	3134.862	4229.109	2909.8	2918.283	2305.394
L	2031.908	2265.048	2558.47	2133.284	2148.682	2422.295	1826.67	2178.083	1917.721
M	2350.212	1809.288	2364.754	2336.832	2567.729	2545.876	2019.517	2122.584	1816.56
N	2946.016	2657.725	2414.411	2328.42	2477.426	2277.444	2527.911	2356.417	2763.158
P	3983.441	3250.514	3071.871	2867.685	2049.734	2130.159	2428.885	2528.464	2827.768
Q	3277.617	2565.734	2878.491	2258.304	2308.102	2823.531	2151.087	2108.122	2746.381
R	3818.29	2275.593	2568.929	2703.072	2750.109	2692.982	2589.072	2294.654	2788.626
S	2771.802	1697.236	1822.459	1604.014	1753.568	1706.53	1835.125	2229.729	2005.1
T	2587.711	2051.486	2790.753	2343.221	2116.084	1762.023	2053.254	2712.079	2624.427
V	1889.454	1782.28	1770.661	1869.951	1926.16	2008.394	1943.365	1518.763	1933.974
W	1910.623	2482.074	2359.241	2865.047	2863.727	3020.667	2801.325	1849.251	1650.885
Y	2053.758	2624.868	2355.601	2576.902	2894.694	3596.445	2750.241	2487.822	2541.712
