# Supplementary material for: Bridging immunogenetics and immunoproteomics: Model positional scanning library analysis for Major Histocompatibility Complex class II DQ in Tursiops truncatus
Source: PLoS One. 2018 Aug 2;13(8):e0201299. doi: 10.1371/journal.pone.0201299 (PMC6072028; doi:10.1371/journal.pone.0201299)
Supplement: S9 Table — The 3,456 sequences derived from amino acids for DQ2-4 were searched for protein matches in the UniProtKB database through the Protein Information Resource (PIR). Sequence matches for proteins originating from reported pathogens in marine mammals are summarized here. Columns listed as (#) refer to numbers identified, or (a) list includes undefined species or proteins. Full details are supplied in S12 Table. (PDF) [file pone.0201299.s011.pdf]

Supp Table 9: Proteins and pathogens identified from MPSLA for DQ 2-4

	DQA 1*02 DQB1*04						
	Organism	#	a	Sequence	Protein	#	a
1	*Acinetobacter sp.*	1	+	VLVAVFVWA	Uncharacterized protein		+
				YLVAFFVLA	Uncharacterized protein		
2	*Actinomyces sp.*			VFVAVAVWY	Uncharacterized protein		+
3	*Ajellomyces sp.*			YFWFFAVLA	ATPase	1	
4	*Aspergillus sp.*	4		YFVFVAVLA	Hexose transporter	1	+
				YLVAFAFLA	Uncharacterized protein		
5	*Bacillus sp.*	2		VLVAVFVLA	Uncharacterized protein	1	+
				YLVAVDVWD	S-layer domain protein		
6	*Bordetella sp.*	1		YLVAFFVLA	Uncharacterized protein		+
7	*Clostridium sp.*	1		VFVFFFVLY	Uncharacterized protein		+
8	*Corynebacterium sp.*	2		VLVFVAVLM	Iron ABC transporter permease	1	+
				VLVAVAVLY	Uncharacterized protein		+
9	*Enterobacter sp.*		+	VLVAFADLD	Diguanylate cyclase with GAF sensor	1	
10	*Enterococcus sp.*	1		VLVAVFVLA	Uncharacterized protein		+
11	*Klebsiella sp.*	1		VLVAFADLD	Diguanylate cyclase	1	
12	*Moraxella sp.*	1		VLVAVAVLM	Uncharacterized protein		+
13	*Mycobacterium sp.*	9	+	YLVAVAVLM	Cobalt transporter	5	+
				VLVAVAVLA	Exopolyphosphatase		
				VLVAVAVWA	Uncharacterized protein		
				VLVDVFDLA	Uncharacterized protein		
				VFVAVADLD	Stage II sporulation protein E		
14	*Nocardia sp.*	4	+	VFVAFDVLY	ATP-dependent DNA ligase	2	
				VFVAVAVLA	Preprotein translocase YidC		
15	*Photobacterium sp.*	2		VFVAVAFLA	BCCT transporter	2	
				VLVAFFVLA	Efflux pump membrane transporter		
16	*Pseudomonas sp.*	3		VLVAVAVLY	Uncharacterized protein	2	+
				VLVAFDVLA	Peroxidase		
				YLVFFAVLY	Polysaccharide biosynthesis protein		
17	*Rhodococcus sp.*	1	+	VLVFVAVLA	Major facilitator superfamily multidrug	2	
				VLVDVDDWA	Phenolphthiocerol synthesis polyketide synthase type I Pks15/1		
18	*Streptococcus sp.*	1		YLVAFAVLA	Uncharacterized protein		
19	*Vibrio sp.*	1		YLVAVFVLM	Sodium dependent phosphate transporter	1	
