# Supplementary material for: Bridging immunogenetics and immunoproteomics: Model positional scanning library analysis for Major Histocompatibility Complex class II DQ in Tursiops truncatus
Source: PLoS One. 2018 Aug 2;13(8):e0201299. doi: 10.1371/journal.pone.0201299 (PMC6072028; doi:10.1371/journal.pone.0201299)
Supplement: S10 Table — The 2,586 sequences derived from amino acids for DQ 1–10 were searched for protein matches in the UniProtKB database through the Protein Information Resource (PIR). Sequence matches for proteins originating from reported pathogens in marine mammals are summarized here. Columns listed as (#) refer to numbers identified, or (a) list includes undefined species or proteins. Full details are supplied in S12 Table. (PDF) [file pone.0201299.s012.pdf]

Supp Table 10: Proteins and pathogens identified from MPSL for DQ 1-10

	DQA1*01 DQB1*10						
	**Organism**	**#**	**a**	**Sequence**	**Protein**	**#**	**a**
1	*Aspergillus sp.*	2		WIGGSAIIL	Uncharacterized protein		+
2	*Bacillus sp.*	4	+	IISSSAAIA	Phage neck	2	+
				IISSSGAIA	Pectin lyase fold/virulence factor		
3	*Campylobacter sp.*	1		IIGSSNIIL	NADH-quinone oxidoreductase subunit D	1	
4	*Candida sp.*	6		VIVSSGAIA	Glutamate 5-kinase	3	+
				VIVSSGAIA	Alpha-glucosidase		
				IIVSSGAIA	Pro1 gamma-glutamyl kinase		
5	*Citrobacter sp.*	1	+	IISGSGIIA	Outer membrane autotransporter	1	+
6	*Clostridium sp.*	2	+	IMSGSAIWA	Uncharacterized protein	2	+
				WISSSNSIL	Hydrolase, alpha/beta domain protein		
				IIVSSAAIL	Bacterial sugar transferase		
7	*Corynebacterium s*	2		IIVSSGSIA	Glutamate 5-kinase	1	
				VIVSSGAIA	Glutamate 5-kinase		
8	*Edwardsiella sp.*	5		WMVGSGAWL	1,4-dihydroxy-2-naphthoate prenyltransferase	1	+
				VISGSAIIA	Uncharacterized protein		
9	*Enterobacter sp.*	2		VIVGSASIA	ABC-type transporter, integral membrane subunit	1	
10	*Enterococcus sp.*	1		IIGGSGIIL	Sugar transport protein	1	
11	*Fusarium sp.*	2		WISGSNAIL	Uncharacterized protein		+
12	*Helicobacter sp.*		+	IIVSSGAIA	Glutamate 5-kinase	1	
13	*Leptospira sp.*	7		VIGSSASIA	ABC-type transport system, ATP binding protein puta	2	
				IIVSSGAIA	Glutamate 5-kinase		
14	*Mycobacterium sp.*	138	+	VIVSSGAIA	Glutamate 5-kinase	2	
				IISGPAAIL	Histidine kinase-, DNA gyrase B-, and HSP90-like ATPase family protein		
15	*Mycoplasma sp.*	1		IISSSGSIL	Uncharacterized protein		+
16	*Nocardia sp.*	1		IIGGSAAWA	Transcriptional regulatory protein ZraR		
17	*Pseudomonas sp.*	16	+	VMGGPNAIL	Bla aacC3-like protein (Fragment)	4	+
				VIVSSGAIA	Oxygen-dependent choline dehydrogenase		
				IIGGSNIIA	Multidrug transporter membrane component/ATP-binding component		
				VIGGSNIIA	Cyclic peptide transporter		
				VIVSSGAIA	Oxygen-dependent choline dehydrogenase		
				VMGSSAAWL	Oxidoreductase		
				VIVSSGAIA	Choline dehydrogenase		
				WIVGSGSWA	Uncharacterized protein		
18	*Rhodococcus sp.*	2	+	VMSSPASIL	Benzoate transporter	1	
19	*Streptococcus sp.*	1		IMGGSAAIL	Acyltransferase	1	
20	*Vibrio sp.*	53	+	VIVSSGAIA	Glutamate 5-kinase	2	+
				VMVSSGAIA	Glutamate 5-kinase		
				IIGGPAAIL	Membrane protein		
				IISGSGIIA	Uncharacterized protein (Fragment)		
				WIGGSAIIL	Maltose O-acetyltransferase		
