# Supplementary material for: Bridging immunogenetics and immunoproteomics: Model positional scanning library analysis for Major Histocompatibility Complex class II DQ in Tursiops truncatus
Source: PLoS One. 2018 Aug 2;13(8):e0201299. doi: 10.1371/journal.pone.0201299 (PMC6072028; doi:10.1371/journal.pone.0201299)
Supplement: S11 Table — The 3,888 sequences derived from amino acids for DQ 1-8were searched for protein matches in the UniProtKB database through the Protein Information Resource (PIR). Sequence matches for proteins originating from reported pathogens in marine mammals are summarized here. Columns listed as (#) refer to numbers identified, or (a) list includes undefined species or proteins. Full details are supplied in S12 Table. (PDF) [file pone.0201299.s013.pdf]

Supp Table 11: Proteins and pathogens identified from MPSL for DQ 1-8

DQA1*01
DQB1*08

	Organism	#	a	Protein		#	a
1	*Acinetobacter sp.*	3	+	LIVVVVLVA	Proteobacterial lipase chaperone family protein	1	+
				LSVVVVLVA	Uncharacterized protein		
				LIVVIVLYW	Uncharacterized protein		
				YIVVISSVA	Uncharacterized protein		
				VIVVVVLVA	Uncharacterized protein		
				VIVLVVLVA	Uncharacterized protein		
2	*Actinomyces sp.*	2		VIYVITSVA	Uncharacterized protein	2	+
				VSVVVTLVA	ABC transporter, permease protein		
3	*Ajellomyces sp.*	1		VIVVVSSVA	Hydroxyacyl-Coenzyme A dehydrogenase type II	1	
4	*Aspergillus sp.*	22		LIILISLYK	Uncharacterized protein	6	+
				LIVLVSLVA	Ditrans,polycis-polyprenyl diphosphate synthase ((2E,6E)-farnesyl diphosphate specific)		
				LSILVSLVA	ABC multidrug transporter, putative		
				LSILVSLYK	Signal recognition particle subunit SRP72		
				LSVLVSLVA	ABC multidrug transporter, putative		
				VIYVISSYA	Uncharacterized protein		
				VMVVVSLVA	Uncharacterized protein		
				VSILVSSYA	DUF829 domain protein (PaxU), putative		
				VSILVVLVA	Amino acid permease		
				YSIVITLVK	Uncharacterized protein		
				YSVLITLVK	Uncharacterized protein		
				YSVVISLYK	Putative Sodium/proline symporter		
				YSVVITLVK	Urea active transporter		
5	*Bacillus sp.*	11	+	LIILITSYA	Protein YfhO	10	+
				LIILIVSVA	BRP/BLH family beta-carotene 15,15'-monooxygenase		
				LIYLISLYA	YndJ-like family protein		
				LMVLISSVA	Uncharacterized protein		
				LSIVISLVA	Quinolone resistance protein		
				LSIVVTSVA	Multidrug ABC transporter permease		
				VIILITLVA	Cytochrome C biogenesis protein		
				VIILVVLYA	Glycerol-3-phosphate ABC transporter permease		
				VIILVVSVA	Carbohydrate ABC transporter membrane protein 1, CUT1 family		
				VIILVVSVA	Transport permease protein		
				VIIVIVLYA	Protein translocase subunit SecY		
				VIVVIVLVA			
				VSVLVTLVA			
				YIIVITLVA			
				YIVLITLVA			
6	*Bordetella sp.*	1	+	LIVLVVLVA	Uncharacterized protein	1	+
				LSVLVSLVA	Fatty-acid--CoA ligase		
7	*Campylobacter sp.*	1	+	LIVLIVLVA	Uncharacterized protein		
8	*Clostridium sp.*	9	+	LSYLIVSYW	D-alanyl-D-alanine carboxypeptidase	7	+

				LSVLVTSYK	Uncharacterized protein		
				LIVLISLYA	Uncharacterized protein		
				LIILIVLVA	Ribonuclease Y		
				LIYLVVLVK	Exfoliative toxin A/B		
				VIVLIVLVW	Inner-membrane translocator		
				VIVVIVLVA	Dipeptide transport system permease protein DppC		
				LIILVVLYA	Uncharacterized protein		
				LIVLVTLYK	Uncharacterized protein		
				VSIVVVLYA	Uncharacterized protein		
				LIVLISLYA	Uncharacterized protein		
				LMYLVSLVA	Uncharacterized protein		
				YMVVVTLVW	ATPase/histidine kinase/DNA gyrase B/HSP90 domain protein		
				VIIVISLYA	Polysaccharide biosynthesis protein		
				LMYLVVLVA	ABC transporter related protein		
				VIVVISLVA	Uncharacterized protein		
9	*Corynebacterium sp.*	6		VIILVSLVA	Virulence factor	2	+
				VIVLVSLVA	Murein biosynthesis protein MurJ		
				LIVLVVLVA	Uncharacterized protein		
				VIVLIVLVW	Uncharacterized protein		
10	*Enterobacter sp.*	1		LIILISSYA	Uncharacterized protein		+
11	*Enterococcus sp.*	4		YIVLIVLVA	Branched-chain amino acid transporter permease	2	+
				LSVVVTLVA	SPFH/Band 7/PHB domain protein		
				LIVLVSLVA	Uncharacterized protein		
				YIVLIVLVA	Uncharacterized protein		
12	*Klebsiella sp.*	1		VSVLITLVW	Ammonium transporter	1	
13	*Leptospira sp.*	10	+	LIILVTSVA	Phospholipase D-nuclease N-terminal domain protein	3	+
				LSILVVSVA	Spirochaetales surface lipoprotein		
				LIILITSVA	Phospholipase		
				LIILVTSVA	Phospholipase_D-nuclease N-terminal		
				LSILISSYA	Uncharacterized protein		
14	*Mycobacterium sp.*	50	+	LIILVVLVA	Transmembrane protein		
				LIIVVTLVA	ABC transporter permease	6	+
				LIVLIVLVA	Uncharacterized protein		
				LIVVIVLVA	ABC transporter permease		
				LIVVVTLVA	Uncharacterized protein		
				LIVVVVLVA	Uncharacterized protein		
				LIYLVVSVA	Uncharacterized protein		
				LMILVVSVK	Iron import ATP-binding/permease protein IrtB		
				LMVLVVLVA	Bacterial extracellular solute-binding s, 5 Middle family protein		
				LSVLIVLVW	Uncharacterized protein		
				LSYVVVLVA	Sulfate ABC transporter inner membrane subunit CysW		
				VIIVITSVK	Murein biosynthesis integral membrane protein MurJ		
				VIVVITLVK	Membrane protein		
				VIVVITSVK	Murein biosynthesis integral membrane protein MurJ		
				VIVVIVLVA	Hypothetical membrane protein		

				VIVVVVLVA	Short-chain fatty acid transporter		
				VMVVVVLVA	Uncharacterized protein		
				VMYVVTSYA	Cytochrome C and Quinol oxidase polypeptide I family protein		
				VSVLVTLYA	MFS transporter		
				VSVVVTLYA	Arsenic transporter		
				YIVLITLVA	MFS transporter, sugar porter family		
				YSVLITSVA	Pyrroline-5-carboxylate reductase (Fragment)		
				YSVLVTSVA	Pyrroline-5-carboxylate reductase		
				YSVLVTSVK	Pyrroline-5-carboxylate reductase		
15	*Mycoplasma sp.*	2		YSILVSSYA	Glucan 1,6-alpha-glucosidase	2	
				LSILVTLVA	FtsX-like permease family protein (Fragment)		
16	*Nocardia sp.*	1		VIVVVVLVA	Uncharacterized protein	1	+
		1		LIVVVTSVW	Putative oxidoreductase		
17	*Photobacterium sp.*	1		VIILVSSYW	Efflux pump membrane transporter BepE	1	
18	*Proteus sp.*	1	+	LIILISLYK	Putative membrane-associated sulfatase	1	
19	*Pseudomonas sp.*	16	+	LIILIVLVA	Glycosyl transferase family 51	9	+
				LIIVITLVA	BCCT transporter		
				LIVVVVLVA	Tetratricopeptide repeat-containing protein		
				LMVLISLVA	Uncharacterized protein		
				LMVVIVLVA	Type 4 fimbrial biogenesis protein FimU		
				LSILIVLYK	Uncharacterized protein		
				LSILVVLVA	Sodium/sulfate symporter family protein		
				VIILVVSYA	Benzoate membrane transport protein		
				VMILVVLVA	Aerotaxis receptor Aer		
				VSVVIVSVA	Glycerol acyltransferase		
				VSVVVTLVA	Uncharacterized protein		
				VSVVVVLVA	FAD:protein FMN transferase		
20	*Rhodococcus sp.*	1		LIVVVVLVA	Histidine kinase	1	
21	*Salmonella sp.*	1		VMILVVLVW	Type VI secretion protein IcmF	1	
22	*Serratia sp.*	5	+	LSVVITLVW	Ammonium transporter	2	
				LSVVVTLVW	Ammonium transporter		
				VSVLITLVW	Ammonium transporter		
				LIVVVVLVA	Protein HflC		
23	*Staphylococcus sp.*	22	+	LIILVVLVK	Cysteine ABC transporter ATP-binding protein	3	+
				LIIVIVLVA	Monofunctional glycosyltransferase		
				LIIVVVLVA	Uncharacterized protein		
				LIVLISSVA	Uncharacterized protein		
				LIVLVVSVK	Thiol reductant ABC exporter, CydC subunit		
				LMILIVSVK	ABC transporter ATPase		
				LMILVVSVK	ABC transporter, ATP-binding protein		
				LMVLIVSVK	Transport ATP-binding protein CydC		
				LMVLVVSVK	Cysteine ABC transporter ATP-binding protein		
				LSYLVTLVA	ABC transporter		
				VSILIVLVA	ABC transporter ATP-binding protein		
				VSVLIVLVA	ABC transporter, ATP-binding protein		
				VSYLVTSVA	Multidrug MFS transporter		

				YSVVIVLVA	Urea transporter		
				YSYVVVLVA	Urea transporter		
24	*Streptococcus sp.*	5	+	LIVVVVLVA	Amino acid permease	2	
				LMILIVSVK	Competence factor transporting ATP-binding/permease protein		
				LMILVVSVK	Transport ATP-binding protein CydC		
				LMVLVVSVK	ABC transporter ATP-binding protein		
				VIILVVSVA	Sugar ABC transporter permease		
				VIVVVTSVA	Arginine:ornithine antiporter		
				YIVVIVLVA	Multidrug ABC transporter permease		
				YIVVVVLVA	Putative ABC transporter, integral membrane protein		
25	*Vibrio sp.*	8		YSILVVLVA	Diguanylate cyclase	4	+
				LIVVIVLVA	Uncharacterized protein		
				LIVVVSLYA	Uncharacterized protein		
				VIYLITSVA	Uncharacterized protein		
				LMIVVTLVA	Putative K+-dependent Na+/Ca+ exchanger-like		
				VIYLISLVA	Uncharacterized protein (Fragment)		
				LSVLISLVA	Multidrug resistance protein NorM		
				LIVVIVLVA	V10 pilin		
26	*Sporothrix sp.*	1		VIVVVVLVW	Uncharacterized protein		+
